# MHC class II expression and potential antigen-presenting cells in the retina during experimental autoimmune uveitis

**DOI:** 10.1186/s12974-017-0915-5

**Published:** 2017-07-18

**Authors:** Deborah A. Lipski, Rémi Dewispelaere, Vincent Foucart, Laure E. Caspers, Matthieu Defrance, Catherine Bruyns, François Willermain

**Affiliations:** 1 Ophthalmology Group, IRIBHM (Institut de Recherche Interdisciplinaire en Biologie Humaine et Moléculaire), Université Libre de Bruxelles (ULB), Erasme Campus, Building C, Room C6.117, 808 Route de Lennik, 1070 Brussels, Belgium; 2 Ophthalmology Department of Erasme Hospital, Université Libre de Bruxelles (ULB), 808 Route de Lennik, 1070 Brussels, Belgium; 3 Ophthalmology Department of CHU Saint-Pierre, 322 Rue Haute, 1000 Brussels, Belgium; 4 Ophthalmology Department of CHU Brugmann, 4 Place Van Gehuchten, 1020 Brussels, Belgium; 5 Interuniversity Institute of Bioinformatics in Brussels, Université Libre de Bruxelles - Vrije Universiteit Brussel, La Plaine Campus, BC building, 6th floor, CP 263, Triomflaan, 1050 Brussels, Belgium

**Keywords:** Autoimmune eye disorders, Inflammation, Blood-retinal barrier, Antigen presentation, Co-stimulatory molecules, Microglia, Macrophages, Ly6C, Transcriptome, RNA-Seq

## Abstract

**Background:**

Controversy exists regarding which cell types are responsible for autoantigen presentation in the retina during experimental autoimmune uveitis (EAU) development. In this study, we aimed to identify and characterize the retinal resident and infiltrating cells susceptible to express major histocompatibility complex (MHC) class II during EAU.

**Methods:**

EAU was induced in C57BL/6 mice by adoptive transfer of autoreactive lymphocytes from IRBP1-20-immunized animals. MHC class II expression was studied by immunostainings on eye cryosections. For flow cytometry (FC) analysis, retinas were dissected and enzymatically digested into single-cell suspensions. Three MHC class II^+^ retinal cell populations were sorted by FC, and their RNA processed for RNA-Seq.

**Results:**

Immunostainings demonstrate strong induction of MHC class II expression in EAU, especially in the inner retina at the level of inflamed vessels, extending to the outer retinal layers and the subretinal space in severely inflamed eyes. Most MHC class II^+^ cells express the hematopoietic marker IBA1. FC quantitative analyses demonstrate that MHC class II induction significantly correlates with disease severity and is associated with upregulation of co-stimulatory molecule expression. In particular, most MHC class II^hi^ cells express co-stimulatory molecules during EAU. Further phenotyping identified three MHC class II^+^ retinal cell populations: CD45^−^CD11b^−^ non-hematopoietic cells with low MHC class II expression and CD45^+^CD11b^+^ hematopoietic cells with higher MHC class II expression, which can be further separated into Ly6C^+^ and Ly6C^−^ cells, possibly corresponding to infiltrating macrophages and resident microglia. Transcriptome analysis of the three sorted populations leads to a clear sample clustering with some enrichment in macrophage markers and microglial cell markers in Ly6C^+^ and Ly6C^−^ cells, respectively. Functional annotation analysis reveals that both hematopoietic cell populations are more competent in MHC class II-associated antigen presentation and in T cell activation than non-hematopoietic cells.

**Conclusion:**

Our results highlight the potential of cells of hematopoietic origin in local antigen presentation, whatever their Ly6C expression. Our work further provides a first transcriptomic study of MHC class II-expressing retinal cells during EAU and delivers a series of new candidate genes possibly implicated in the pathogenesis of retinal autoimmunity.

**Electronic supplementary material:**

The online version of this article (doi:10.1186/s12974-017-0915-5) contains supplementary material, which is available to authorized users.

## Background

The major histocompatibility complex (MHC) is a set of cell surface proteins divided into two major groups respectively known as class I and class II molecules, which play a fundamental role in adaptive immunity. While MHC class I is ubiquitously expressed by almost all cells, MHC class II is mostly expressed by antigen-presenting cells (APCs) such as monocytes, macrophages, and dendritic cells. These cells are involved in external antigen (Ag) processing and antigenic peptide presentation in the context of MHC class II to CD4+ T helper (T_h_) cells. Full T_h_ cell activation occurs when the peptide-MHC class II complex interacts with the T cell receptor (TCR), in the presence of signals delivered by the interaction of co-stimulatory molecules such as CD40, CD80, and CD86 on the APC and their ligands on T cells.

Interestingly, expression of MHC class II is not strictly restricted to immune cells. It has been demonstrated that non-professional APCs are capable of inducible MHC class II expression, Ag presentation, and even effective T cell reactivation [1, 2]. Aberrant expression of MHC class II by non-professional APCs from targeted organs and subsequent presentation of auto-Ags is now considered to be an important mechanism in the pathogenesis of autoimmune disease processes, including those affecting the eye [3, 4].

Experimental autoimmune uveitis (EAU) is a model of organ autoimmunity in the eye. EAU is mediated by activated T_h_ cells, which are believed to be central in the pathogenesis of human non-infectious uveitis as well [5, 6]. Immunization with IRBP in adjuvant context leads to priming of autoreactive T cells in peripheral lymphoid organs and polarization into T_H_1 and T_H_17 cells. These activated T_h_ cells then home to the eye, where they induce blood-retinal barrier (BRB) breakdown and subsequent massive recruitment of diverse inflammatory leukocytes from the circulation [7]. It has been shown that while the first activated T cells enter the eye by chance, regardless of their specificity for retinal or non-retinal Ags, only retina-specific T cells induce EAU [8, 9]. This leads to the conclusion that EAU induction requires T_h_ cell restimulation by in situ Ag recognition. However, the main targets in IRBP-induced EAU are the photoreceptors, which are not believed to express MHC class II. In this context, a tremendous series of works have tried to determine which cell types are responsible for intra-ocular Ag presentation during EAU. Both resident retinal cells [10] and infiltrating hematopoietic cells [11] have been proposed, with inconsistent results.

In this study, we aimed to identify and characterize the retinal resident and infiltrating cells susceptible to express MHC class II during adoptive transfer (AT) EAU.

## Methods

### Reagents and animals

Interphotoreceptor retinoid-binding peptide (IRBP) 1–20 (GPTHLFQPSLVLDMAKVLLD), representing residues 1–20 of human IRBP, was synthesized by New England Peptide (Gardner, MA, USA). Pertussis toxin (PTX) and complete Freund’s adjuvant (CFA) were purchased from Sigma-Aldrich (Bornem, Belgium). Pathogen-free female C57BL/6 mice (6 to 10 weeks old), purchased from Janvier (Genest St Isle, France) were housed at the animal facilities in accordance with the European guidelines. Animal treatment conformed to the ARVO Statement for the Use of Animals in Ophthalmic and Vision Research. All cells were cultured in RPMI 1640 medium supplemented with 25 mM HEPES, 10% fetal bovine serum, 1% l-glutamine, 1% sodium-pyruvate, 100 IU/ml penicillin, 100 g/ml streptomycin, 5.10^−5^ M β-mercaptoethanol in a 5% CO2, and 95% humidity incubator.

### Classical and adoptive transfer models of experimental autoimmune uveitis

Classical EAU was induced by immunization of naive C57BL/6 mice with a subcutaneous injection in each hind leg of 50 μl of a mixture containing 500 μg/100 μl IRBP peptide 1–20 in a 1:1 emulsion with CFA enriched with 2.5 mg/ml of heat-inactivated mycobacterium tuberculosis. All animals simultaneously received an intraperitoneal injection of 1 μg of PTX. Adoptive transfer EAU was induced by AT of autoreactive lymphocytes following the protocol of Shao H et al. [12]. Briefly, animals were immunized as mentioned above. Twelve days after immunization, mice were euthanized and their spleen and draining lymph nodes dissected and dissociated. Spleen cell suspensions were enriched in T lymphocytes through passage on nylon wool fiber columns, then pooled with total lymph node cells and re-stimulated in vitro with IRBP1-20 (1 μg/ml). After 2 days in culture, cells were injected intraperitoneally into naive C57BL/6 mice (5 × 10^6^ cells/mouse).

### Disease grading

A clinical grading was performed at day 14 or day 21 after disease induction. Mice were anesthetized by a 50-μl intraperitoneal injection of a Rompun (0.2%) and Ketalar (20 mg/ml) mixture. The pupils were dilated with tropicamid (5 mg/ml) and phenylephrine (1.5 mg/ml), and the eyes were examined under the slit-lamp of a surgical microscope (Zeiss, Göttingen, Germany) by using a cover slip coated in a viscoelastic gel (synthetic polymer of acrylic acid 2 mg/g, Vidisic, Tramedico, Belgium) and positioned on the cornea. The clinical grading was performed independently by two ophthalmologists, based on a system adapted from Xu et al. [13]. Briefly, vitritis, optic neuropathy, retinitis, and vasculitis were separately scored in each eye, from 0 (no disease) to 4 (highly severe disease) with half-point increments and averaged to generate the clinical score of the eye on a scale from 1 to 4. The clinical score attributed to one mouse corresponds to the mean of the scores of the two eyes.

### Immunohistology

#### Immunofluorescence stainings on retinal cryosections

At day 14 or 21 after disease induction, the eyes were collected, prefixed for 6 h at 4 °C in PFA (paraformaldehyde) 4% and sucrose 3%, and then put in three successive baths containing 5, 10, and 18% sucrose in PBS, respectively, for 24 h each. The entire eyes were embedded in OCT medium (Sakura, Antwerp, Belgium) and cut in 16-μm-thick frozen sections using a cryostat (CM3050S Leica). The MOM (mouse-on-mouse) Basic Kit (Vector Laboratories, Labconsult, Brussels) was used to prevent high background staining. Cryosections were fixed with PFA 4% for 15 min and blocked in TBS (Tris 10 mM, NaCl 0.9%, pH 7.6) supplemented with MOM IgG blocking solution and Triton 0.3% for 2 h. Sections were incubated overnight with the following primary antibodies, alone or in different combinations as indicated in the results: anti-MHC class II (rat, 1/200; BD Biosciences, Erembodegem, Belgium), anti-GFAP (mouse, 1/500; Millipore, Brussels, Belgium), anti-endoglin (goat, 3/500; BD Biosciences), anti-CD31 (goat, 1/200, R&D systems, Abingdon), and anti-IBA1 (goat, 1/100, Abcam, Cambridge, UK), diluted in TBS supplemented with MOM kit protein concentrate. After three washings in TBS, the sections were incubated in the dark for 1 h30 with species-specific secondary antibodies coupled to different fluorochromes, as indicated in data, then with Hoechst to stain the nuclei (Invitrogen, Gent, Belgium). After several washings, the sections were mounted in Glycergel (Dako, Agilent Technologies, Diegem, Belgium) supplemented with 2.5% Dabco (Sigma-Aldrich). Pictures of immunostainings were acquired using an AxioImager Z1 microscope equipped with an AxioCamMR camera (Carl Zeiss, Inc.) using the z-stack mode of the Axiovision acquisition software. Z-stacks were processed using the Imaris deconvolution software. Differential interference contrast (DIC) images were recorded on an AxioImager Z1 (Zeiss) upright widefield microscope using a Plan Apochromat 20x\0.8 NA (Zeiss) air objective combined with the corresponding EC PN (II) prism slider (Zeiss) under the objective and polarizing filters in the light path.

#### Immunofluorescence stainings on retinal wholemount preparations

At day 21 after disease induction, the eyes were collected and immediately immersed in PFA 4% for 1 h at 4 °C. The eyes were then dissected in ice-cold PBS: the anterior segment of the globe, crystalline lens, and vitreous were removed and the retina was carefully peeled from the retinal pigment epithelium. Whole retinas were fixed in 70% ethanol for 1 h, rinsed three times in PBS (10 min each), blocked with a solution containing 3% milk and 3% bovine serum albumin in PBS for 1 h and incubated with anti-MHC class II (rat, 1/200; BD Biosciences, Erembodegem, Belgium) and anti-endoglin (goat, 1/200; BD Biosciences) antibodies in a PBS-BSA1% solution overnight at room temperature. The retinas were rinsed three times in PBS (20 min each) and incubated sequentially for 1 h with each secondary antibody. After three final washings in PBS (20 min each), each retina was flattened by radial incisions and mounted with Vectashield antifade mounting medium with DAPI (Labconsult, Brussels).

### Preparation of retinal single-cell suspensions

Mice were sacrificed at day 14 or 21 after disease induction, and the eyes immediately enucleated. Eyes were carefully hemisected in HBSS buffer containing penicillin/streptomycin 1%, with surgical scissors under a surgical microscope. Retinal tissue was isolated and rinsed in HBSS medium. The two retinas of each mouse were pooled and cut into small pieces before enzymatic digestion in 3 ml HBSS containing 1.6 mg/ml Liberase (Roche, Vilvoorde, Belgium) and 0.1 mg/ml DNase I (Sigma-Aldrich) at 37 °C for 45 min. Cell dissociation was stimulated by pipetting every 15 min. Cells were washed with DMEM/10% FBS and filtered through a 40-μm cell strainer to obtain a single-cell suspension [14]. The yield was approximately 2 to 3 million retinal cells per mouse.

### Fluorescence-activated cell sorting (FACS) analysis

Retinal single-cell suspensions were tested by flow cytometry (FC) for their membrane expression of MHC class II (I-A/I-E), CD45 (pan-leucocyte marker), CD11b (myeloid cell marker), Ly6C (monocyte/macrophage marker), CD31 (endothelial cell marker), CD40, CD80 and CD86 (co-stimulatory molecules), and F4/80 (pan-macrophage marker) using specific antibodies (BD Biosciences) coupled to different fluorochromes. For Lipocalin 2 (Lcn2) and Cysteinyl Leukotriene Receptor 1 (Cysltr1), unconjugated primary antibodies (from R&D systems and Abcam, respectively) and species-specific secondary antibodies coupled to Alexa488 were used. Cells were incubated with the relevant antibodies for 20 min at 4 °C, washed and resuspended in FACS buffer. For Lcn2 intracytoplasmic staining, cells were first incubated with membrane antibodies then permeabilized with Cytofix/cytoperm fixation/permeabilization solution (BD Biosciences) before incubation with the anti-Lcn2 antibody. Live cells were gated with Hoechst 1/4000 and debris and doublets were excluded (the complete gating strategy is illustrated in Additional file 1: Figure S1). Up to one million total cells per sample were analyzed on a LSR-Fortessa flow cytometer using the CellQuest Software (BD Biosciences). Isotypes and *Fluorescence minus one* (FMO) controls were used for accurate gating. Compensations were performed using BD CompBeads (BD Biosciences).

### Analysis of retinal cell gene expression

#### Purification of different MHC class II^+^ populations

Three weeks after AT, mice were sacrificed and retinal single-cell suspensions prepared as described above. Cells were stained with PE-labeled anti-MHC class II, PECy7-labeled anti-CD45, FITC-labeled anti-CD11b and APC-labeled anti-Ly6C antibodies. MHC class II^+^CD45^+^CD11b^+^Ly6C^+^ cells (referred to as *Plus*), MHC class II^+^CD45^+^CD11b^+^Ly6C^−^ cells (referred to as *Minus*), and MHC class II^+^CD45^−^CD11b^−^Ly6C^−^ (referred to as *non-hematopoietic* or NH) were separately sorted by preparative FC using a FACSAria with the FACSDiva Software (BD). Due to the low cell number obtained from each mouse (around 1000 *Plus* cells), three mice were pooled to generate each sample. Cells were sorted directly in lysis buffer, vortexed for 30 s and flash frozen in liquid nitrogen. The purity of the sorted cell populations was evaluated by FC re-analysis of sorted cells (Additional file 2: Figure S2).

#### RNA extraction

RNA extraction was performed using the MiRNeasy MicroKit (Qiagen) according to the manufacturer’s recommendations and a DNase step to avoid DNA contamination. RNA quality was assessed using the Agilent 2100 Bioanalyzer with RNA 6000 Pico kit (Agilent Technologies).

#### RNA processing and RNA sequencing

Indexed cDNA libraries were prepared using the Ovation Single Cell RNAseq system (Nugen). The multiplexed libraries were loaded and sequences were produced using a TruSeq PE cluster and SBS-kit on a HiSeq 1500 (Illumina). Approximately 25 million paired-end reads/sample were mapped against the mouse reference genome (NCBI Build 37/UCSC mm9) using STAR software to generate read alignments for each sample. Expression levels were quantified using the featureCounts [15] tool and the UCSC RefSeq gene annotation as a reference (exons only, genes as meta features). Differential analysis between the groups was performed using the EdgeR package (quasi-likelihood F-tests). Normalized expression levels were estimated using the EdgeR rpm function and converted to log_2_ FPKM (fragments per kilobase of exon per million mapped reads) after resetting low FPKMs to 1. To perform blind clustering analysis, genes were selected based on the overall variance between samples (independently of their category), by keeping only the 30 most variant ones. Functional analysis was performed using the DAVID web-based functional annotation tool [16].

### Statistical analysis

Statistical analysis was performed using Kruskal-Wallis, ANOVA, Tukey post-hoc multiple comparisons test, and Student’s *t* test. Only *p* values <0.05 were considered statistically significant.

## Results

### Immunofluorescence analysis of MHC class II expression during EAU

**Fig. 1:**
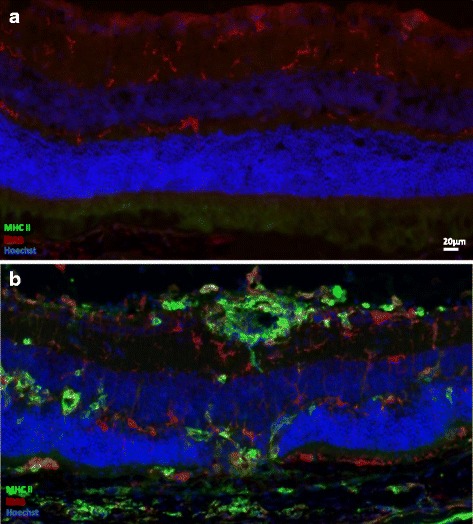
MHC class II retinal expression is highly induced during EAU. Three weeks after adoptive transfer, eye cryosections were prepared and stained for MHC class II (*green*) and IBA1 (*red*) detection. Naive eyes were used as control. Cell nuclei were stained with Hoechst (*blue*). Each picture was chosen as representative of an experiment conducted on three or more animals. **a** Naive retina. **b** Adoptive transfer EAU retina (grade 3.5)

Naive or EAU eyes enucleated 21 days after AT were prepared for MHC class II expression analysis by immunofluorescence (IF). As compared to naive mice, MHC class II expression in the retina is much more intense and extended during EAU (Fig. 1). Most MHC class II-bearing cells co-stain with IBA1, a marker classically used to identify macrophages and microglia (quantification of simple and double-stained cells is provided in Additional file 3: Figure S3). Whereas in naive mice, IBA1-expressing microglial cells are confined to the inner retina (Fig. 1a), during severe uveitis microglial radial processes extending from the outer plexiform layer into the outer nuclear layer (ONL), with concurrent photoreceptor death and ONL thinning (Fig. 1b). We also analyzed the expression of MHC class II during classical EAU at disease peak (day 21) as well as during AT EAU induction (day 14). Representative images are included in Additional file 3: Figure S3 and Additional file 4: Figure S4. No difference was observed regarding MHC class II expression in classical EAU compared to AT EAU matched for disease grade. Not all animals show signs of uveitis at day 14 after AT. We processed the eyes from three independent animals with signs of disease and three independent animals with normal fundus appearance for IF stainings. When no signs of EAU appearing in the fundus, IF images were quite similar to what are observed in naïve eyes, with very discrete expression of MHC class II around the optic nerve and at the ora serrata (data not shown). When signs of disease were present, IF images were very evocative of stainings at day 21.

#### MHC class II expression in the neural retina

**Fig. 2:**
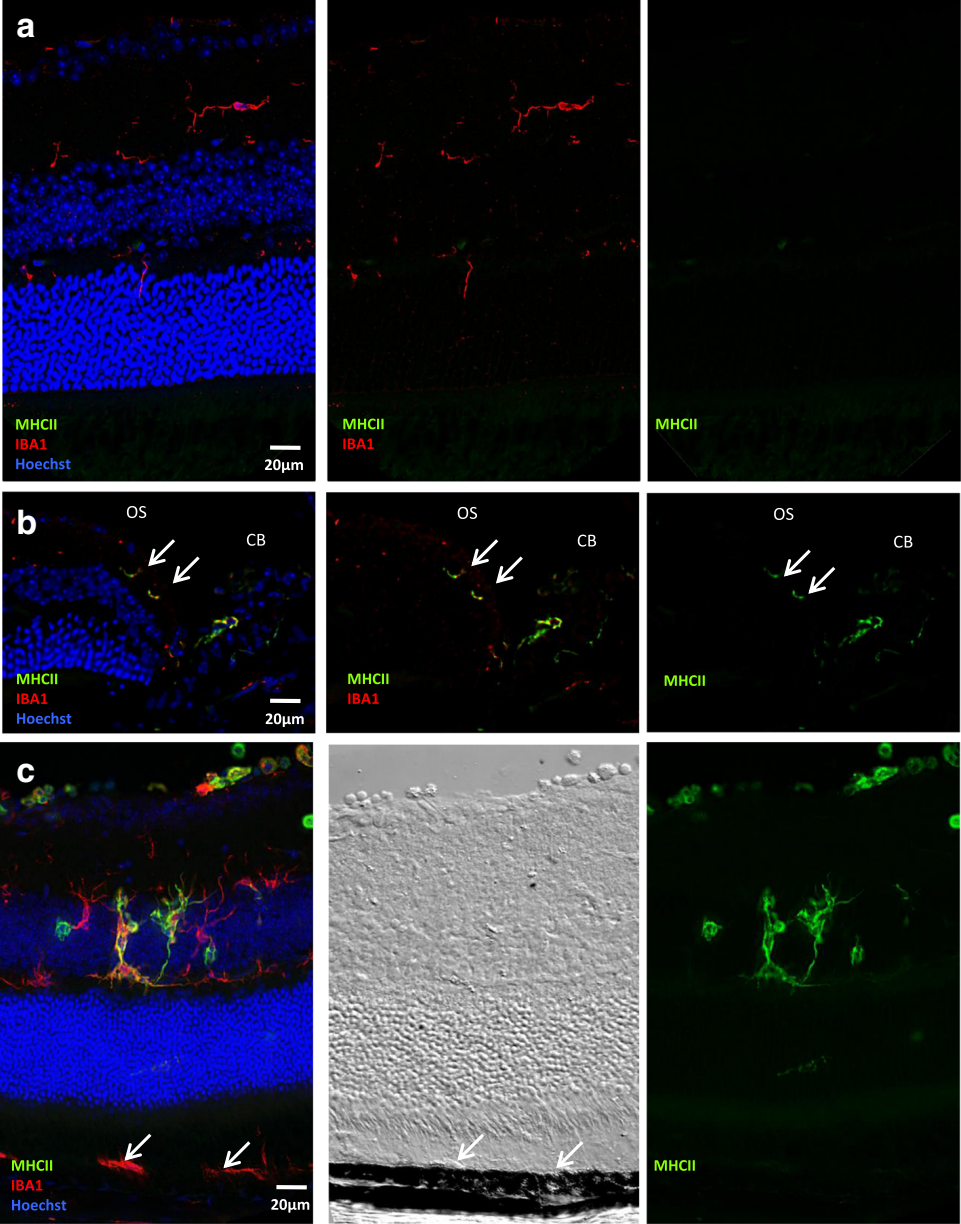
MHC class II expression in the neural retina. Three weeks after adoptive transfer, EAU eye cryosections were prepared and stained for MHC class II (*green*) and IBA-1 (*red*) detection. Naive eyes were used as control. Cell nuclei were stained with Hoechst (*blue*). Each picture was chosen as representative of an experiment conducted on three or more animals. The differential interference contrast (DIC) image was added to localize the RPE more precisely. **a** Expression of MHC class II and IBA1 in the naive retina. **b** Expression of MHC class II and IBA1 in the naive retina at the ora serrata (OS, *arrows*), adjacent to the ciliary body (CB). **c** Expression of MHC class II and IBA1 during EAU and corresponding DIC image (*arrows* point to IBA^+^ cells at the level of the RPE layer)

In naive eyes, most neural retina is devoid of MHC class II^+^ cells (Fig. 2a). Weak expression by dendriform cells is occasionally found around inner retinal vessels, especially surrounding the optic nerve (data not shown). MHC class II expression is systematically found at the level of the ciliary body, extending to the ora serrata in the form of rare dendriform cells, co-stained with IBA1 (Fig. 2b, white arrows). During EAU, microglial cells adopt an activated morphology with larger cell bodies and amoeboid processes (Fig. 2c) and MHC class II expression develops in dendriform cells mostly spanning from the inner plexiform layer to the outer plexiform layer and eventually invading the ONL. It is noticeable that IBA1 staining is more extensive than MHC class II staining and that not all IBA1^+^ cells express MHC class II (Fig. 2c and Additional file 3: Figure S3D). Surprisingly, IBA1 is expressed at the level of the retinal pigment epithelium (RPE) layer; although, DIC imaging suggests that this expression corresponds to IBA1+ hematopoietic cells adhering to the RPE rather than to the RPE cells themselves (Fig. 2c).

#### MHC class II expression at the level of the RPE and on cells infiltrating the vitreous

**Fig. 3:**
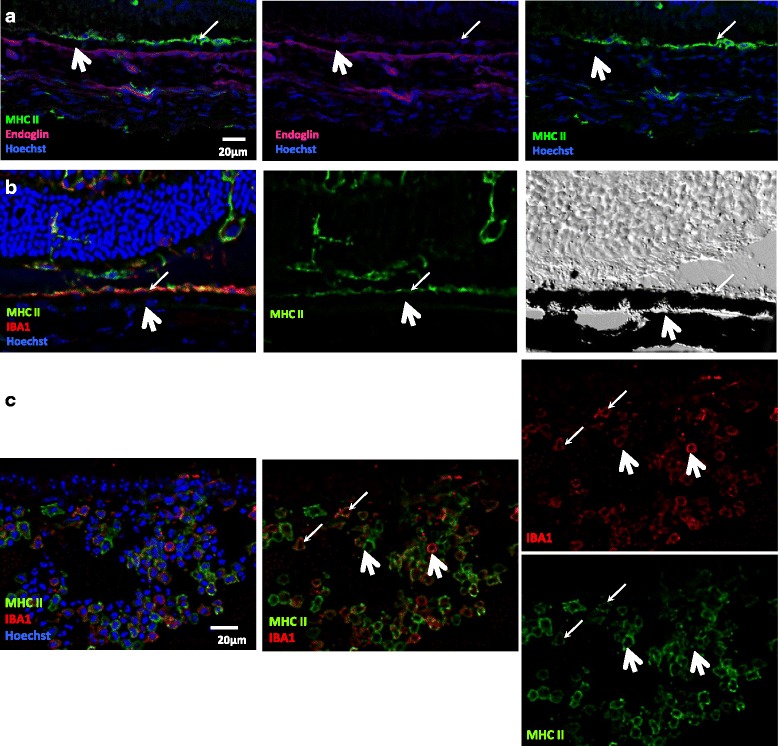
MHC class II is expressed at the level of the RPE layer and by cells infiltrating the vitreous. Three weeks after adoptive transfer, EAU eye cryosections were prepared and stained for MHC class II (*green*), IBA1 (*red*), or endoglin (*magenta*) detection. Cell nuclei were stained with Hoechst (*blue*). Each picture was chosen as representative of an experiment conducted on three or more animals. The DIC image was added to localize the RPE more precisely. **a** MHC class II and endoglin expression in the RPE region. *Thin arrows* point to the RPE layer, and *thick arrows* indicate choroidal vessels stained with endoglin. **b** MHC class II and IBA1 expression in the RPE region and corresponding DIC image. *Thin arrows* point to the RPE layer, and *thick arrows* indicate the choroid. **c** MHC class II and IBA1 expression by cells infiltrating the vitreous. *Thin arrows* indicate cells positive for both markers, and *thick arrows* point to cells positive for only one marker

Sparse expression of MHC class II is found at the level of the RPE layer (Fig. 3a, b, thin arrows), right above the choriocapillaris (stained with endoglin, Fig. 3a, thick arrows), which generally appears to co-localize with IBA1 (Fig. 3b, thin arrows) and could thus represent infiltrating cells. Here again, the DIC image seems to point out an expression by hematopoietic cells adjacent to the RPE rather than by RPE cells.

MHC class II expression is found on most cells infiltrating the vitreous (Fig. 3c). Major co-staining with IBA1 likely identifies those cells as macrophages, considering that no microglial cell is found in the vitreous.

#### MHC class II expression at the level of inflamed vessels

**Fig. 4:**
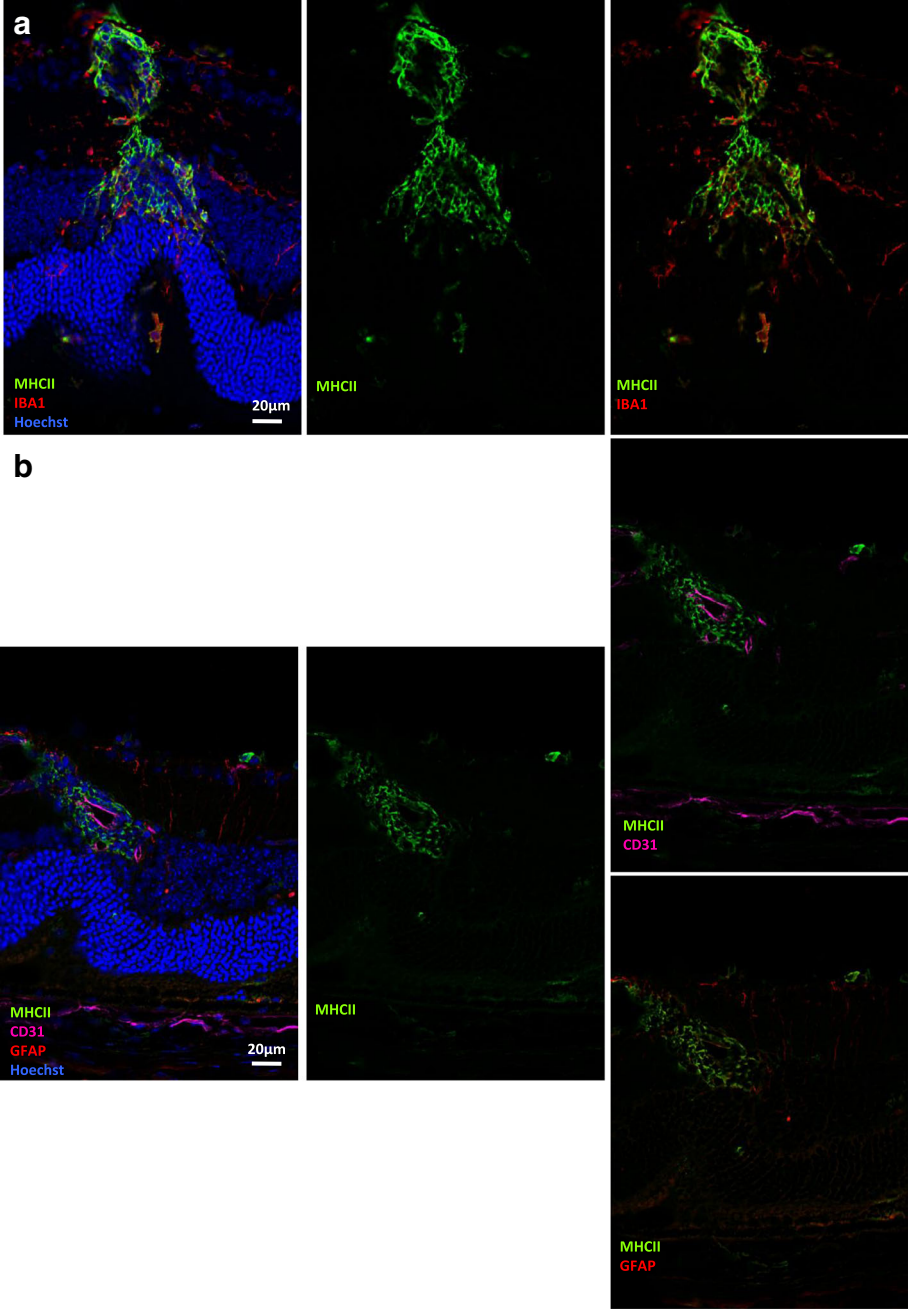
MHC class II expression on inflamed vessels. Three weeks after adoptive transfer, eye cryosections were prepared and stained for MHC class II (*green*), IBA1 (*red*) or GFAP (*red*), and CD31 (*magenta*) detection. Cell nuclei were stained with Hoechst (*blue*). Each picture was chosen as representative of an experiment conducted on three or more animals. **a** MHC class II and IBA1 expression at the level of vasculitis. **b** MHC class II, CD31, and GFAP expression at the level of vasculitis

A dense network of MHC class II^+^IBA1^+^ double-positive cells is found around vasculitis lesions (Fig. 4a). However, at the perivascular level, it is particularly difficult to differentiate microglia from macrophages, since the latter infiltrate the retina mainly through inflamed vessels. Indeed, many round-shaped cells co-expressing MHC class II and IBA1 are associated with blood vessels from which they seem to be getting out into the retina (Figs. 1b and 4). We could not detect a clear co-staining of MHC class II with CD31^+^ endothelial cells or GFAP^+^ glial cells in the diseased retina, suggesting that, in this model, even during uveitis, endothelial and glial cells mostly do not express MHC class II (Fig. 4b). As shown more precisely in Fig. 1b, MHC class II expression seems to follow a “trans-retinal” pattern, high expression of MHC class II around inflamed retinal vessels being often found at the same level as MHC class II expression in outer retinal layers and on inflammatory cells infiltrating the vitreous.

### Quantification and phenotyping of retinal cells expressing MHC class II

**Fig. 5:**
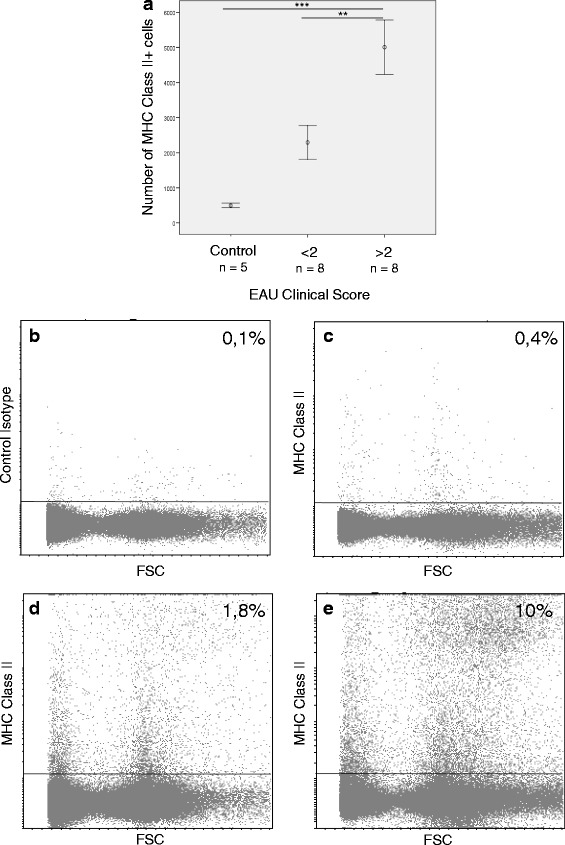
MHC class II expression correlates to EAU severity. Three weeks after adoptive transfer, retinas were carefully dissected, cut into small pieces, and dissociated by incubation with Liberase DL and DNase I at 37 °C for 45 min. Naive eyes were used as control. The single-cell suspensions, excluding dead cells (DAPI+) were analyzed by flow cytometry for MHC II expression using fluorochrome-conjugated specific antibodies. For Figures B to E, data are from 1 representative mouse out of 21 independent mice (5 control, 8 low-score EAU and 8 high-score EAU mice). **a** Number of MHC class II-expressing cells (normalized per 1 million analyzed retinal cells) for different EAU clinical scores. Data represented: mean ± SEM, ANOVA, and Tukey post-hoc multiple comparisons test, **p < 0.01, ***p < 0.001. **b** Naive eye with control isotype. **c** MHC class II expression in the naive retina. **d** MHC class II expression in the retina during moderate uveitis (clinical grade 1). **e** MHC class II expression in the retina during severe uveitis (clinical grade 4)

During inflammation, massive recruitment of inflammatory cells renders difficult the interpretation of IF images, especially around blood vessels. To further precise the nature and number of MHC class II-expressing cells, retinal single-cell suspensions were characterized by FC. Data from Fig. 5 show that the number of MHC class II^+^ cells is very discriminative between healthy and EAU mice (*p* value <0.001). Moreover, a significantly higher number of MHC class II^+^ cells is found in high-grade (grade ≥2) uveitis compared to low-grade (grade <2) uveitis (Fig. 5a) (*p* value <0.01). Indeed, as illustrated by representative mice, we observed around 0.4% MHC class II-expressing cells in the healthy retina (Fig. 5c), 1.8% in EAU grade 1 eyes (Fig. 5d), and up to 10% in grade 4 eyes (Fig. 5e).

#### Co-stimulatory molecule expression is induced on some MHC class II^+^ cells during EAU

**Fig. 6:**
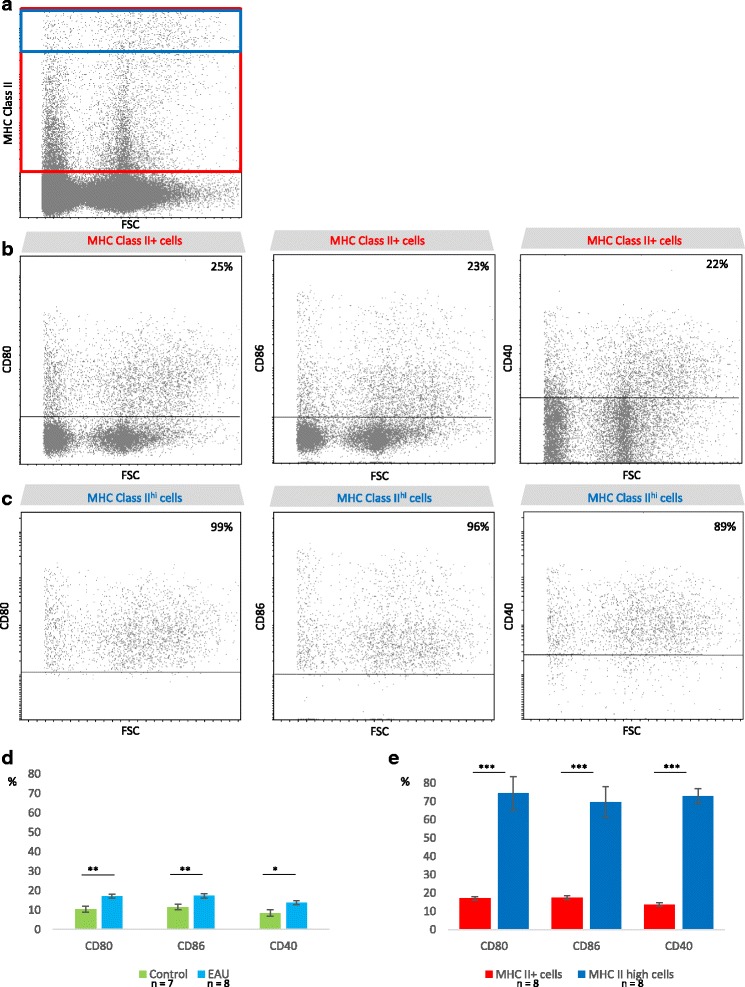
MHC class II^+^ cells upregulate co-stimulatory molecule expression during EAU. Three weeks after adoptive transfer, the retinas were carefully dissected, cut into small pieces, and dissociated by enzymatic digestion. Naive eyes were used as control. The single-cell suspensions, excluding dead cells (DAPI+) were analyzed by flow cytometry for MHC class II, CD40, CD80, and CD86 expression using fluorochrome-conjugated specific antibodies. Data are from one representative mouse (clinical grade 3.75) out of four independent experiments involving eight independent EAU animals. Statistical analysis was performed using *t* tests, *p value <0.05, **p value <0.01, ***p < 0.001. **a** Gating on MHC class II^+^ (*red gate*) and MHC class II^hi^ (*blue gate*) cells. **b** Expression of co-stimulatory molecules by MHC class II^+^ cells. **c** Expression of co-stimulatory molecules by MHC class II^hi^ cells. **d** Histogram representation of the mean percentage of co-stimulatory molecule expression by MHC class II^+^ cells in naive versus EAU retinas. Mean ± SEM (unpaired *t* test). **e** Histogram representation of the mean percentage of co-stimulatory molecule expression by MHCII^hi^ versus MHC class II^+^ cells during EAU. Mean ± SEM (paired samples *t* test)

To assess their potential as effective APCs, we next investigated whether retinal MHC class II^+^ cells also express co-stimulatory molecules such as classically CD80, CD86, and CD40 during EAU. Naive retinas were used as control. During EAU, MHC class II^+^ cells (Fig. 6a, red gate) display upregulated expression of CD80, CD86, and CD40 (Fig. 6b), compared to healthy retinal cells (Fig. 6d). Interestingly, a significantly higher proportion of MHC class II^hi^ cells (Fig. 6a, blue gate) compared to MHC class II^+^ cells express co-stimulatory molecules during EAU (Fig. 6c, e). Kinetics of co-stimulatory molecule expression by MHC class II^+^ cells during classical EAU and AT EAU are illustrated in Additional file 5: Figure S5.

#### Phenotypic characterization of MHC class II-expressing cells during EAU

**Fig. 7:**
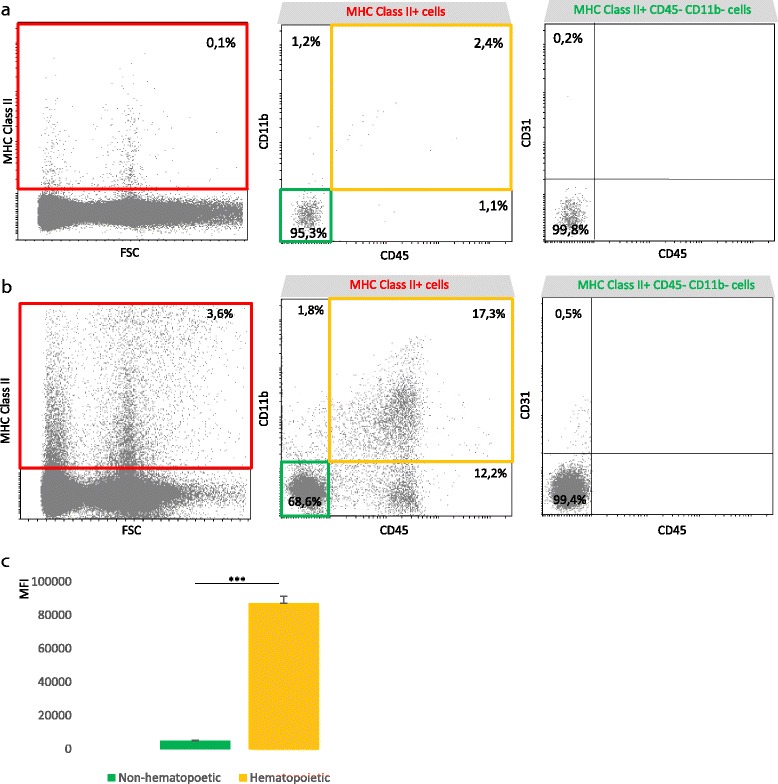
During EAU, both hematopoietic and non-hematopoietic but mostly not endothelial cells are susceptible to express MHC class II. Three weeks after adoptive transfer, the retinas were carefully dissected, cut into small pieces, and dissociated by enzymatic digestion. Naive eyes were used as control. The single-cell suspensions, excluding dead cells (DAPI+) were analyzed by flow cytometry for MHC class II, CD45, CD11b, and CD31 expression using fluorochrome-conjugated-specific antibodies. Data are from one representative mouse out of three independent experiments involving four naive and eight EAU-independent animals. **a** Naive retina. *Left*: total retinal cells. *Middle*: gated on MHC class II^+^ cells (*red gate*). *Right*: gated on non-hematopoietic MHC class II^+^ CD45^−^CD11b^−^ cells (*green gate*). **b** Adoptive transfer uveitis (clinical grade 3.75). *Left*: total retinal cells. *Middle*: gated on MHC class II^+^ cells (*red gate*). *Right*: gated on non-hematopoietic MHC class II^+^ CD45^−^CD11b^−^ cells (*green gate*). **c** Histogram representation of the mean fluorescence intensity (MFI) for MHC class II expression by CD45^+^CD11b^+^ hematopoietic cells (*orange gate*) and CD45^−^CD11b^−^ non-hematopoietic cells (*green gate*) during EAU. Mean ± SEM, Student’s *t* test, ***p < 0.001

In naive retina, the few cells that express MHC class II are at 95% CD45^−^CD11b^−^ non-hematopoietic cells (Fig. 7a, green gate). Among those MHC class II^+^ non-hematopoietic cells, only 0.2% express CD31 (Fig. 7a). As shown in Fig. 7b, uveitis development is associated with a clear increase in MHC class II^+^ hematopoietic cells co-expressing CD45 and CD11b (orange gate). However, there is almost no change in the percentage of MHC class II^+^ non-hematopoietic cells that express CD31, confirming that endothelial cells mostly do not express MHC class II in this model of EAU. Interestingly, even during EAU, a large proportion of MHC class II^+^ cells are non-hematopoietic, but as shown in Fig. 7c, their level of MHC class II expression is much lower than in hematopoietic cells.

**Fig. 8:**
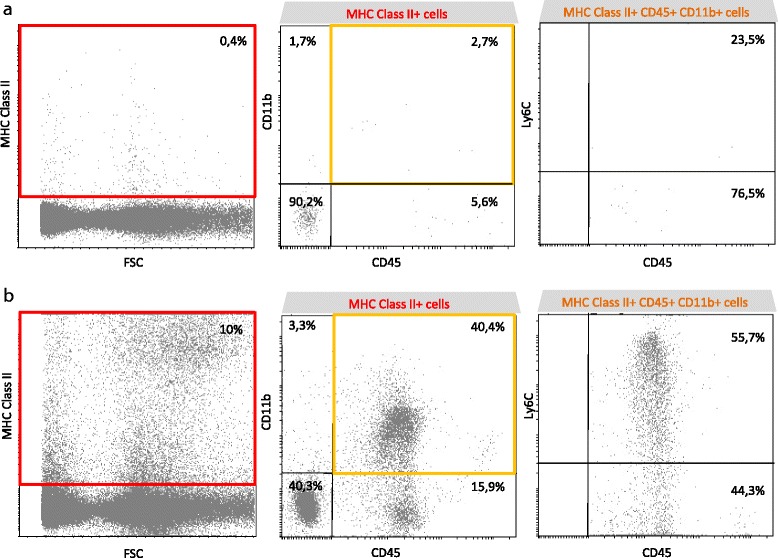
During EAU, retinal hematopoietic MHC class II^+^ cells comprise both Ly6C^+^ and Ly6C^−^ cells. Three weeks after adoptive transfer, retinas were carefully dissected, cut into small pieces, and dissociated by enzymatic digestion. Naive eyes were used as control. The single-cell suspensions, excluding dead cells (DAPI+) were analyzed by flow cytometry for MHC class II, CD45, CD11b, and Ly6C expression using fluorochrome-conjugated-specific antibodies. Data are from one representative mouse out of five independent experiments involving 7 naive and 12 EAU-independent animals. **a** Naive retina. *Left*: total retinal cells. *Middle*: gated on MHC class II^+^ cells (*red gate*). *Right*: gated on hematopoietic MHC class II^+^ CD45^+^CD11b^+^ cells (*orange gate*). **b** Adoptive transfer uveitis (clinical grade 4). *Left*: total retinal cells. *Middle*: gated on MHC class II^+^ cells (*red gate*). *Right*: gated on hematopoietic MHC class II^+^ CD45^+^CD11b^+^ cells (*orange gate*)

To get further insight into the nature of MHC class II^+^ hematopoietic cells, we investigated the expression of the Ly6C marker, suspected to be expressed by infiltrating macrophages and not by resident microglia [17]. Figure 8a shows that in naive retina, the few MHC class II^+^ hematopoietic cells (orange gate) are mainly Ly6C^−^. During uveitis, the high increase in MHC class II^+^ hematopoietic cells leads to almost equal proportions of Ly6C^+^ and Ly6C^−^ cells (Fig. 8b).

FC analysis of MHC class II^+^ cells in the retina was also performed in the two disease models at different timepoints (Additional file 6: Figure S6). When matched for disease grade, no significant difference was evidenced between different models and timepoints as concerns % of cells expressing MHC class II, % of hematopoietic cells among MHC class II^+^ cells, MFI for MHC class II expression among hematopoietic or non-hematopoietic MHC class II^+^ cells, and % of Ly6C^+^ cells among hematopoietic MHC class II^+^ cells (all *p* values >0.05).

### Purification and RNA-Seq analysis of different MHC class II-expressing retinal cell populations

We next wanted to dissect the role in Ag presentation of those Ly6C^−^ (resident?) and Ly6C^+^ (infiltrating?) cells during EAU. Unfortunately, the number of cells in each subpopulation of MHC class II^+^ cells in the retina was far too low for in vitro functional testing of lymphocyte activation and proliferation. We thus used RNA-Seq analysis to compare the gene expression of separately sorted MHC class II^+^ hematopoietic cells, further divided into Ly6C^+^ cells (referred to as *Plus*) and Ly6C^−^ cells (referred to as *Minus*) and MHC class II^+^ non-hematopoietic cells that expressed neither CD45/CD11b nor Ly6C (referred to as *non-hematopoietic* or *NH*) (three independent experiments, three mice pooled to generate each sample).

**Fig. 9:**
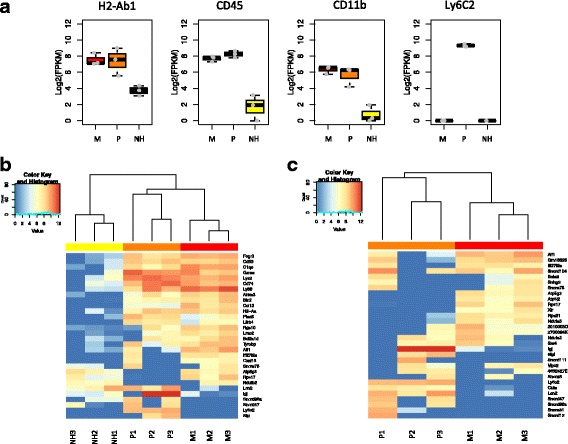
Transcriptome analysis confirms the existence of different retinal cell populations expressing MHC class II during EAU. Three weeks after adoptive transfer, retinal single-cell suspensions were analyzed by flow cytometry and sorted into three different cell populations: MHC class II^+^CD45^+^CD11b^+^Ly6C^+^ (*Plus* or P), MHC class II^+^CD45^+^CD11b^+^Ly6C^−^ (*Minus* or M), and MHC class II^+^CD45^−^CD11b^−^Ly6C^−^ cells (*Non-hematopoietic* or NH). Each sample corresponds to a pool of three mice. **a** Cell population-dependent expression of the markers used for cell sorting. Data represented: boxplot of normalized mRNA expression levels (presented as Log_2_FPKM). **b** Heatmap and blind clustering analysis displaying the gene expression profiles for the 30 genes with the highest variance among all samples. Each row represents an individual gene and each column a sample. **c** Heatmap and blind clustering analysis displaying the gene expression profiles for the 30 genes with the highest variance among *Plus* and *Minus* samples. Each row represents an individual gene and each column a sample

Figure 9a confirms that the sorting strategy was very effective as all markers used for cell sorting are correctly expressed at the mRNA level. We next generated a heatmap displaying the level of expression of the 30 most variant transcripts among all 9 samples. Blind bioinformatics analysis successfully leads to a clear clustering of all samples into three distinct populations, with major differences being found between *Plus* or *Minus* cells on the one side and *NH* cells on the other side (Fig. 9b). In addition, a similar procedure restricted to the six hematopoietic cell samples still allows a clear clustering of *Plus* and *Minus* cell populations (Fig. 9c).

**Fig. 10:**
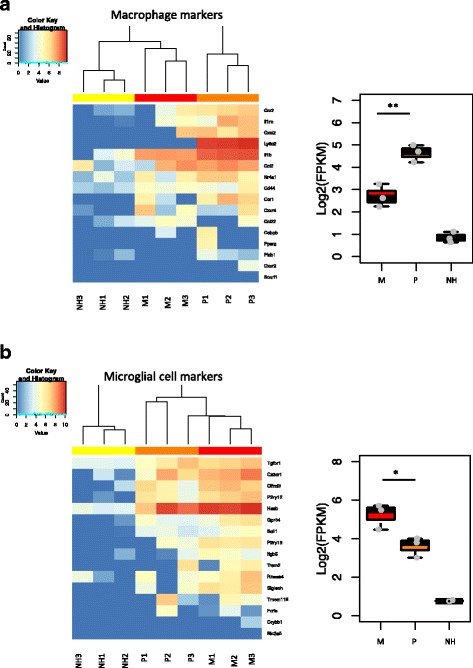
*Plus* cells are enriched in macrophage makers while *Minus* cells preferentially express microglial cell markers. Three weeks after adoptive transfer, retinal single-cell suspensions were analyzed by flow cytometry and sorted into three different cell populations: MHC class II^+^CD45^+^CD11b^+^Ly6C^+^ (*Plus* or P), MHC class II^+^CD45^+^CD11b^+^Ly6C^−^ (*Minus* or M), and MHC class II^+^CD45^−^CD11b^−^Ly6C^−^ cells (*Non-hematopoietic* or NH). **a** *Left*: heatmap displaying the gene expression profiles for genes known to be expressed by macrophages. Blind clustering analysis leads to clear separation of the nine samples into three cell populations. *Right*: boxplot representation of the mean expression of macrophage markers by each cell population. **b** *Left*: heatmap displaying the gene expression profiles for genes known to be expressed by microglia. Blind clustering analysis leads to clear separation of the nine samples into three cell populations. *Right*: boxplot representation of the mean expression of microglial cell markers by each cell population. Each row represents an individual gene and each column a sample from a pool of three mice. Statistical analysis was performed using *t* tests, *p value <0.05, **p value <0.01

Next, in order to explore whether Ly6C expression or non-expression allows distinction of macrophages from microglia, markers specific for macrophages [18, 19] or microglia [20] were selected from the literature. Figure 10a, b illustrates heatmaps displaying the relative mRNA levels of those genes in our samples. A relative but significant enrichment in macrophage markers is observed in the *Plus* population and conversely for microglial markers in the *Minus* population. However, some macrophage cell markers are also expressed by *Minus* cells and some microglial cell markers are expressed by *Plus* cells.

Direct transcriptome comparison between *Plus* and *Minus* cells reveals 17 genes significantly modulated between the 2 cell populations (FDR ≤0.05) (Table 1). Tables 2 and 3 show the 30 most significantly regulated genes between *Plus* and *NH* and between *Minus* and *NH* cells, respectively.

**Table 1:** Gene signature of MHC class II^+^ *Plus* versus *Minus* cells

Gene symbol	Gene name	Log_2_FC (P/M)	FDR
Igj	Immunoglobulin joining chain	8.6779562	2.29E-11
Ly6c2	Lymphocyte antigen 6 complex, locus C2	12.30121239	2.92E-09
Slfn4	Schlafen 4	15.5668286	1.75E-06
Lcn2	Lipocalin 2	7.312076677	3.37E-05
Slpi	Secretory leukocyte peptidase inhibitor	12.73813718	5.33E-05
Ccdc126	Coiled-coil domain containing 126	−8.341365207	0.000225043
Atp5g3	ATP synthase, H+ transporting, mitochondrial F0 complex, subunit C3 (subunit 9)	−11.69540624	0.000280756
Fsd1l	Fibronectin type III and SPRY domain containing 1-like	−6.703008314	0.000462131
Dnajc12	DnaJ heat shock protein family	−14.11777559	0.000618267
Rbm8a	RNA binding motif protein 8a	−11.28615955	0.001526552
Fat3	FAT atypical cadherin 3	−14.08641235	0.003575335
Col3a1	Collagen, type III, alpha 1	14.61654737	0.003584831
Akap17b	A kinase (PRKA) anchor protein 17B	−10.53057236	0.009607167
Cysltr1	Cysteinyl leukotriene receptor 1	−14.65367542	0.009752316
Sgip1	SH3-domain GRB2-like (endophilin) interacting protein 1	−6.380242524	0.011651515
Pou2af1	POU domain, class 2, associating factor 1	9.055182034	0.019402858
Dnm1	Dynamin 1	−12.9307709	0.050431759

**Table 2:** Gene signature of MHC class II^+^ *Plus* versus *Non-hematopoietic* cells

Gene symbol	Gene name	Log_2_FC (P/NH)	FDR
Ly6c2	Lymphocyte antigen 6 complex, locus C2	11.87357484	6.66E-09
Rac2	RAS-related C3 botulinum substrate 2	11.3730173	6.66E-09
Igj	Immunoglobulin joining chain	6.717543668	1.03E-07
Ncr1	Natural cytotoxicity triggering receptor 1	15.15445896	1.24E-07
Clec5a	C-type lectin domain family 5, member a	14.40445716	1.29E-07
Lilrb4	Leukocyte immunoglobulin-like receptor, subfamily B, member 4A	11.01126519	3.08E-07
Fli1	Friend leukemia integration 1	11.46778052	3.13E-07
Ly86	Lymphocyte antigen 86	6.798033679	3.68E-07
Gzma	Granzyme A	6.705242775	2.05E-06
Ctss	Cathepsin S	5.568780709	2.10E-06
Il18r1	Interleukin 18 receptor 1	12.72237638	2.10E-06
Gimap6	GTPase, IMAP family member 6	15.15864883	2.10E-06
Ctla4	Cytotoxic T-lymphocyte-associated protein 4	14.67103293	2.31E-06
Bin2	Bridging integrator 2	7.565458144	2.31E-06
Cd74	CD74 antigen (invariant polypeptide of MHC, class II antigen-associated)	6.334079696	2.59E-06
Ccdc126	Coiled-coil domain containing 126	−9.776447913	2.62E-06
Fcgr3	Fc receptor, IgG, low affinity III	6.739932279	2.62E-06
Cd53	CD53 antigen	6.362290092	2.80E-06
CD45	Protein tyrosine phosphatase, receptor type, C	6.186653535	2.91E-06
Fsd1l	Fibronectin type III and SPRY domain containing 1-like	−8.081824945	2.91E-06
F4/80	Adhesion G protein-coupled receptor E1	8.087894562	2.91E-06
Psd4	Pleckstrin and Sec7 domain containing 4	14.6851914	2.91E-06
Arhgdib	Rho, GDP dissociation inhibitor (GDI) beta	6.696195491	2.91E-06
Bank1	B cell scaffold protein with ankyrin repeats 1	15.15703742	4.08E-06
Pou2af1	POU domain, class 2, associating factor 1	15.22372718	4.65E-06
Fyb	FYN binding protein	6.232404471	6.35E-06
Vsir	V-set immunoregulatory receptor	8.022010319	6.95E-06
Slpi	Secretory leukocyte peptidase inhibitor	12.58889492	8.50E-06
Lyz2	Lysozyme 2	5.875787951	9.14E-06
Anxa3	Annexin A3	6.959651951	9.14E-06

**Table 3:** Gene signature of MHC class II^+^ *Minus* versus *Non-hematopoietic* cells

Gene aymbol	Gene name	Log_2_FC (M/NH)	FDR
Ly86	Lymphocyte antigen 86	8.243860053	5.61E-09
Ctss	Cathepsin S	6.957996729	9.43E-09
Bin2	Bridging integrator 2	9.235207039	2.73E-08
Rac2	RAS-related C3 botulinum substrate 2	10.60734368	2.73E-08
Clec5a	C-type lectin domain family 5, member a	15.17887896	2.73E-08
F4/80	Adhesion G protein-coupled receptor E1	9.972224185	3.90E-08
Fcgr3	Fc receptor, IgG, low affinity III	7.775892583	1.20E-07
Gatm	Glycine amidinotransferase (L-arginine:glycine amidinotransferase)	8.65338182	2.18E-07
Anxa3	Annexin A3	8.397158434	2.55E-07
Ncr1	Natural cytotoxicity triggering receptor 1	14.1982847	3.02E-07
C1qc	Complement component 1, q subcomponent, C chain	7.38704385	4.49E-07
Fli1	Friend leukemia integration 1	10.50996433	1.65E-06
Ccl12	Chemokine (C-C motif) ligand 12	15.2104167	2.66E-06
Hexb	Hexosaminidase B	6.279744813	2.88E-06
Fyb	FYN binding protein	6.513964387	3.63E-06
Cx3cr1	Chemokine (C-X3-C motif) receptor 1	6.581332934	3.77E-06
Ctla4	Cytotoxic T-lymphocyte-associated protein 4	14.1739945	4.06E-06
Cd53	CD53 antigen	6.217529713	5.05E-06
Fcgr1	Fc receptor, IgG, high affinity I	10.93059649	5.90E-06
IBA1	Allograft inflammatory factor 1	7.685685163	7.39E-06
Kctd12	Potassium channel tetramerisation domain containing 12	6.343559988	7.75E-06
Lmo2	LIM domain only 2	7.844870456	8.06E-06
Lilrb4	Leukocyte immunoglobulin-like receptor, subfamily B, member 4A	9.104873913	8.23E-06
Psd4	Pleckstrin and Sec7 domain containing 4	13.93584229	8.46E-06
Il18r1	Interleukin 18 receptor 1	11.54035827	8.91E-06
Mpeg1	Macrophage expressed gene 1	5.934098653	9.09E-06
Csf1r	Colony stimulating factor 1 receptor	6.563851162	1.14E-05
Itgb2	Integrin beta 2	7.391669553	1.16E-05
Arhgdib	Rho, GDP dissociation inhibitor (GDI) beta	6.250951528	1.23E-05
C1qb	Complement component 1, q subcomponent, beta polypeptide	6.508090307	1.42E-05

*FDR* false discovery rate

**Fig. 11:**
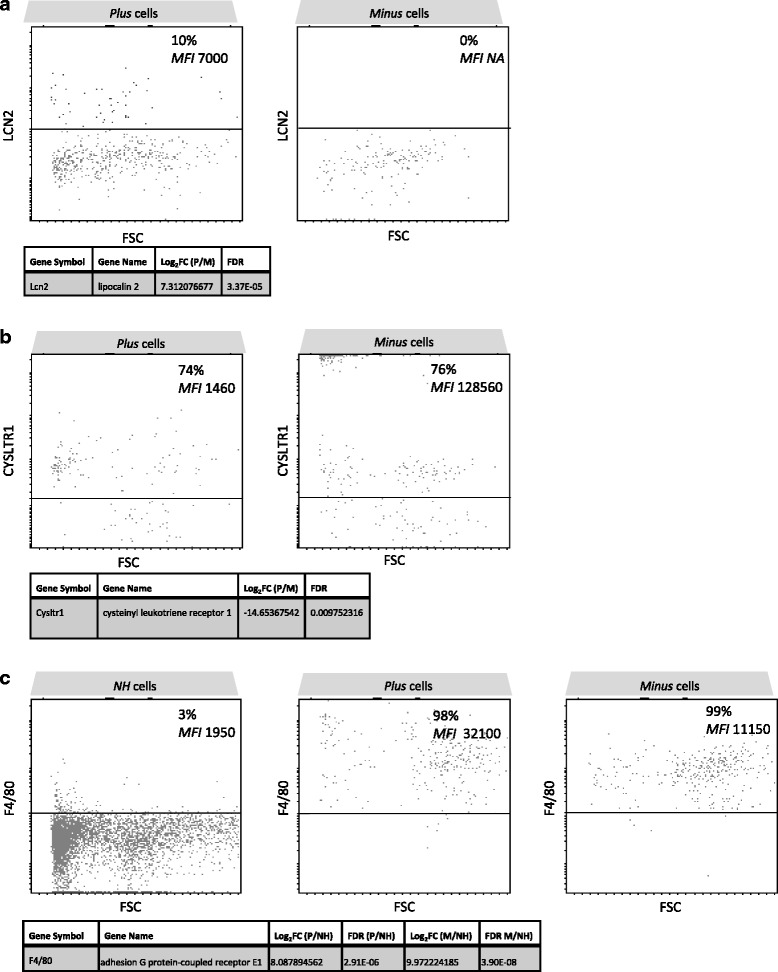
Validation of RNA-Seq data at the protein level. At day 21, after disease induction, the retinas were carefully dissected, cut into small pieces, and dissociated by enzymatic digestion. The single-cell suspensions, excluding dead cells (DAPI+) were analyzed by flow cytometry for MHC class II, CD45, CD11b, Ly6C, and either F4/80, LCN2, or Cysltr1 expression using fluorochrome-conjugated specific antibodies. Data are from one representative mouse out of two independent experiments involving six independent EAU animals. Data represented: percentage of cells expressing the marker and mean fluorescence intensity (MFI). Below dot plots, respective gene symbols, gene names, Log_2_FC and FDR for each marker are indicated. **a** Lcn2 differential expression by *Plus* and *Minus* cells. **b** Cysltr1 differential expression by *Plus* and *Minus* cells. **c** F4/80 differential expression by *NH*, *Plus*, and *Minus* cells

The expression of three representative genes were validated at the protein level by FC. According to RNA-Seq data, Lcn2 expression is higher in *Plus* than that in *Minus* cells (Table 1). Cysltr1, conversely, is downregulated in *Plus* compared to *Minus* cells (Table 1). Finally, F4/80 is upregulated in *Plus* and *Minus* cells compared to *NH* cells (Tables 2 and 3). The percentage of cells expressing each gene product and respective MFI among the three different cell populations are illustrated in Fig. 11. Although involving small numbers of cells, data were highly reproducible among the six independent animals analyzed in two separate experiments.

Finally, functional analysis with the David web-based tool reveals that both hematopoietic cell populations play a more important role in Ag processing and presentation in association with MHC class II and other pro-inflammatory functions than *NH* cells (Table 4, FDR <0.05). No clear functional difference emerges between *Plus* and *Minus* cells (data not shown).

**Table 4:** Functional analysis of RNA-Seq data

Log_2_FC (P/NH)	GO terms	Log_2_FC (M/NH)
45.2	Antigen processing and presentation of exogenous peptide antigen via MHC class II	67.9
12.4	Positive regulation of T cell proliferation	15.9
12.0	Phagocytose, engulfment	15.5
5.7	Inflammatory response	6.1

## Discussion

EAU is a classical model of autoimmunity in which retinal Ag-specific T_H_ cells drive the development of retinal inflammation. Local recognition of a retinal Ag is known to be required for T_H_ cell reactivation and an important unresolved question is to determine which cells are responsible for in situ MHC class II expression and Ag presentation. The retina was long thought to be entirely devoid of MHC class II, as part of the immune privilege. However, in line with our data, some studies have highlighted faint MHC class II expression in the naive retina, as well as an increased expression of MHC class II during uveitis [21]. Despite years of research, great controversies remain on the role of MHC class II expression by different cell types in uveitis development. Our strategy to investigate this topic was to focus our studies on retinal MHC class II-expressing cells in a natural, non-transgenic, model of uveitis.

In this work, we demonstrate a strong upregulation, both in extent and intensity, of MHC class II expression in the retina during EAU. We also show that MHC class II induction significantly correlates with disease severity and is associated with higher co-stimulatory molecule expression, particularly on most MHC class II^hi^ cells. We further identify three MHC class II^+^ retinal cell populations: CD45^−^CD11b^−^ cells of non-hematopoietic origin with low MHC class II expression and CD45^+^CD11b^+^ cells of hematopoietic origin expressing higher levels of MHC class II, which can be further separated into Ly6C^+^ and Ly6C^−^ cells. Transcriptome analysis of the three sorted populations leads to a clear sample clustering with some enrichment in macrophage markers and microglial cell markers in Ly6C^+^ and Ly6C^−^ cells, respectively. Finally, functional annotation analysis reveals no major functional differences between Ly6C^+^ and Ly6C^−^ cells, both hematopoietic cell populations playing a more important role in Ag processing and presentation in association with MHC class II and in T cell activation than non-hematopoietic cells.

Our FC data provide evidence for a non-hematopoietic cell population that expresses MHC class II both in naïve neuro-sensory retina and during uveitis, although to a lower level than cells of hematopoietic origin. However, the nature of these cells could not be identified by IF. Indeed, we did not find strong MHC class II expression on astrocytes nor Müller cells. In agreement, Zhang et al. did not find MHC class II expression on astrocytes nor Müller cells during uveitis in an IFN-γ-induced model of ocular inflammation in rats [22]. In contrast, Jiang G et al. showed that retinal astrocytes express MHC class II during EAU in B10RIII mice [1]. Similarly, expression of MHC class II by RPE cells during uveitis was already described in both mice [23] and humans [24] more than 20 years ago. However, although our IF data show some MHC class II expression at the level of the RPE, both contrast phase images and co-staining with IBA1 seem to indicate that this expression is attributable to infiltrating hematopoietic cells. Such MHC class II expression by IBA1^+^ subretinal cells has actually been described in rd8 mutant mice [25]. As concerns MHC class II expression by endothelial cells, neither immunostainings nor FC data demonstrate a clear expression. Surprisingly, both the presence [4, 26] and absence [11, 22] of MHC class II expression on endothelial cells have been described in the literature. This controversy can partially be explained by the use of different models and techniques. To further investigate this point, we also performed MHC class II staining on retinal wholemounts, confirming the presence of MHC class II^+^ cells around retinal vessels, mostly in the shape of dendriform cells ensheathing the vessel (Additional file 7: Figure S7). This is in agreement with the work of Xu et al., who observed no expression of MHC class II by vascular endothelial cells in EAU retinal wholemounts [27]. Our data thus somehow question the fact that endothelial cells play a prominent role in Ag presentation to T_h_ cells during EAU, even though those cells are the central element of the inner BRB and probably the first inner retinal cells encountered by autoreactive lymphocytes.

Recent and quite provocative data suggest that even neuronal cell types are capable of both constitutive and inducible MHC class II expression. Tonade et al. have recently shown that photoreceptor cells produce inflammatory mediators that stimulate leukocytes during diabetic retinopathy [28]. Another argument in favor of the possible role of photoreceptors in retinal inflammation is that opsin-driven SOCS1 overexpression mitigates EAU development [29]. Vagaska et al. further demonstrated that MHC class II is expressed in a subpopulation of human neural stem cells and on neurons, at least in vitro [30]. Finally, Charles et al. have even observed MHC class II expression on retinal neurons such as photoreceptors, during toxoplasma infection [31]. Yet unvalidated data from our lab suggest that at least part of the *NH* MHC class II^dim^ cell population might be composed of photoreceptors. Rods represent 80% of retinal cells. Contamination of the transcriptome of different retinal cell types by photoreceptor genes has been described by different groups, even when using a highly stringent sorting method relying on transgenic mouse lines in which different retinal cells are marked with fluorescent proteins [32, 33]. This contamination thus represents a possible limitation of our study and further investigation of this point is clearly needed.

MHC class II expression in the absence of appropriate co-stimulatory signals has been associated with induction of T cell apoptosis or anergy [34]. Our FC quantitative analyses of retinal single-cell suspensions demonstrate that MHC class II induction during EAU is associated with upregulation of co-stimulatory molecule expression. Furthermore, almost all cells expressing high levels of MHC class II display co-stimulatory molecules, probably indicating higher APC potential. Unfortunately, due to the low absolute number of MHC class II-expressing cells, we were not able to perform extensive phenotypic nor functional analyses of those co-stimulatory molecule-expressing cells. In the literature, data relative to in vivo retinal expression of co-stimulatory molecules are sparse. Tissue-resident cells with inducible MHC class II such as glia or vascular endothelium were reported to express CD80 and CD86 among other co-stimulatory signals under certain conditions and alter responses locally [35]. To our knowledge, only one study has described the expression of B7.1 (CD80) and B7.2 (CD86) in the eye at different timepoints during experimental autoimmune anterior uveitis, showing that both co-stimulatory molecules are expressed during the disease and downregulated with remission [36]. Besides, blockade of B7/CD28 [36] and disruption of CD40/CD40L interactions [37] were shown to inhibit EAU induction.

Within the retina, both resident and infiltrating cells have been reported to express MHC class II. In the normal retina, the only cells of hematopoietic origin were thought to be yolk sac-derived microglia. However, at least in the central nervous system, recent works also suggest the existence of perivascular macrophages of the same embryologic origin [38, 39]. During EAU, the retina is further invaded by infiltrating macrophages which express MHC class II [40]. Considering that MHC class II upregulation is also a hallmark of reactive microglia, the increase in MHC class II^+^CD45^+^CD11b^+^ cells can thus correspond either to the activation and replication of microglia or to invading macrophages. Moreover, although it has recently become clear that monocyte-derived macrophages recruited during inflammation and normal tissue-resident microglia have distinct developmental origins (bone marrow and yolk sac, respectively), a phenotypic overlap exists, with sharing of pan-macrophage markers such as IBA1 and CD11b. No research protocols allow perfect discrimination between those two cell types. Irradiation chimerism or parabiosis induces bias and/or technical limitations [19]. As concerns the transgenic CCR2^rfp^:Cx3cr1^gfp^, mouse model used to discriminate between CX3CR1-GFP resident microglia and CCR2-RFP infiltrating monocyte-derived macrophages [41], not all monocyte-derived macrophages express CCR2 and they are also susceptible to express CX3CR1 [42]. Within the genetically unmodified mouse model of uveitis we used, some works have found that the level of CD45 expression defines CD45^intermediate^ and CD45^high^ populations, corresponding to microglia and recruited macrophages, respectively [43]. However, it has also been shown that activated microglia upregulate CD45 [44, 45] and that differentiation of monocytes into macrophages may be associated with downregulation of CD45 [46], sometimes to levels that make the two cell populations indistinguishable [47]. Accordingly, our FC data did not provide evidence for a clear CD45^intermediate^ versus CD45^high^ population among MHC class II^+^ cells. Another option was to choose Ly6C expression to further discriminate microglia from macrophages among MHC class II^+^ cells of hematopoietic origin. Indeed, several works showed that Ly6C is expressed mainly by infiltrating macrophages and not by microglia [18, 48]. We thus isolated Ly6C-positive (*Plus*) and Ly6C-negative (*Minus*) cells among MHC class II^+^CD45^+^CD11b^+^ hematopoietic cells and used transcriptome analysis to get insight into the nature and role of each cell type. Our bioinformatics analysis confirms to some extent the validity of Ly6C as a discriminative marker; although, some macrophage markers are expressed by Ly6C^−^ cells and some microglial cell markers are expressed by Ly6C^+^ cells. Indeed, it is known that not all macrophages express Ly6C [49], and even that Ly6C expression is downregulated when monocytes migrate into tissues and differentiate into macrophages [46]. This could explain why macrophage markers are more significantly enriched in Ly6C^+^ cells than microglial cell markers in Ly6C^−^ cells, since the Ly6C^−^ population may contain Ly6C^−^ macrophages.

Only 17 genes were found to be significantly regulated between *Plus* and *Minus* cells, including Ly6C2. Among those differentially regulated genes, four are known inflammatory genes: Slfn4, Lcn2, Slpi, and Cysltr1. Three of those genes are upregulated in the *Plus* population: Slfn4 is known to be upregulated during macrophage activation [50], while Lcn2 and Slpi are both upregulated in the experimental autoimmune encephalomyelitis (EAE) model [51, 52]. Lcn2 levels were even shown to be increased in the aqueous humor of patients with idiopathic acute anterior uveitis compared to controls [53]. Cysltr1 is upregulated in the *Minus* population and is known as a pro-inflammatory protein produced mainly by cells of the innate immune system including monocytes/macrophages [54]. Furthermore, two other significantly regulated genes, Sgip1 and Dnm1, are known for a role in the phagocytosis process. Besides, we found that Pou2af1, a gene still poorly described in the literature, is preferentially expressed by macrophages over microglia, in agreement with Gonzalez-Pena et al. [55].

Functional analysis reveals no major difference between *Plus* and *Minus* cells. Controversy exists regarding potential functional differences between microglia and macrophages in autoimmune diseases of the central nervous system. Wlodarczyk et al. demonstrated that, in the EAE model, a subpopulation of CD11c^+^ microglial cells are as effective as CD11c^+^ infiltrating cells in inducing proliferation of myelin oligodendrocyte glycoprotein-immunized T_H_ cells [2]. Contrariwise, Yamasaki et al. highlighted many differences between the two cell types in the EAE model, macrophages being highly phagocytic and inflammatory whereas microglial cells had a globally suppressed metabolism [19]. As concerns EAU, Gregerson et al. demonstrated that CD45^+^ cells isolated from quiescent retina have little ability to present Ag, even when LPS-activated [56]. In our experimental settings, both Ly6C^+^ and Ly6C^−^ hematopoietic cell populations seem to play an important and comparable role in Ag presentation and T cell activation, when compared to non-hematopoietic cells.

## Conclusion

Our results highlight the potential of cells of hematopoietic origin in local Ag presentation, whatever their Ly6C expression. Our work further provides a first transcriptomic study of MHC class II-expressing retinal cells during EAU and delivers a series of new candidate genes possibly implicated in the pathogenesis of retinal autoimmunity.

## Additional files

Additional file 1: **Figure S1.** Complete gating strategy for flow cytometry experiments. Retinas were carefully dissected, cut into small pieces, and dissociated by incubation with Liberase DL and DNase I at 37 °C for 45 min. The single-cell suspensions were analyzed by flow cytometry. A. FSC versus SSC representation of the total cell population. The first gate was placed to exclude debris (P1). B. Within P1, Hoechst staining was used to exclude dead cells (P2). C. Within P2, doublets were excluded based on SSC (P3). D. Within P3, doublets were also excluded based on FSC (P4). All subsequent analyses were performed on cells gated in P4. (PPTX 106 kb)

Additional file 2: **Figure S2.** Purity of the sorted cell populations. Three weeks after adoptive transfer, retinal single-cell suspensions were analyzed by flow cytometry and sorted into three different cell populations, MHC class II^+^CD45^+^CD11b^+^Ly6C^+^ (*Plus*), MHC class II^+^CD45^+^CD11b^+^Ly6C^−^ (*Minus*), and MHC class II^+^CD45^−^CD11b^−^Ly6C^−^ (*NH*) cells. Each sample was sorted from a pool of three mice. Sorted cells were then re-analyzed by flow cytometry to assess the purity of cell sorting. Although hampered by very low cell numbers due to death or adherence to tube of many cells between the two analyses, this figure illustrates the purity among live MHC class II^+^ cells. A. Sorted *NH* cells. B. Sorted *Minus* cells. C. Sorted *Plus* cells are too rare to allow reliable re-analysis (most re-analyzed *Plus* cells are found in the gate containing dead cells and debris). (PDF 354 kb)

Additional file 3: **Figure S3.** MHC class II retinal expression is highly induced during classical EAU and adoptive transfer EAU, both during induction and at disease peak. Eye cryosections were stained for MHC class II (green) and IBA1 (red) detection 21 days after classical EAU induction (B), 14 days (C) or 21 days after adoptive transfer (AT) (D). Naive eyes were used as control (A). In each picture, quantification was made with the co-staining module of the Imaris 7.3 software. Each cell was counted individually. Results are expressed as the percentage of IBA1^+^ or MHCII^+^ single positive cells and IBA1^+^MHCII^+^ double-positive cells among the total of single and double-positive cells. The DIC image was added to better localize the RPE. A. MHC class II expression in naïve eyes. B. MHC class II expression during classical EAU at day 21. C. MHC class II expression during AT EAU at day 14. D. MHC class II expression during AT EAU at day 21. (PPTX 3600 kb)

Additional file 4: **Figure S4.** MHC class II expression in the retina during classical EAU. Three weeks after immunization, eye cryosections were prepared and stained for MHC class II (green) and IBA1 (red) or endoglin (magenta) detection. Cell nuclei were stained with Hoechst (blue). Each picture was chosen as representative of an experiment conducted on six or more animals. A. MHC class II and IBA1 expression. B. MHC class II and endoglin expression. (PPTX 7276 kb)

Additional file 5: **Figure S5.** Kinetics of co-stimulatory molecule expression by MHC class II± cells during classical EAU and adoptive transfer EAU. Fourteen or 21 days after disease induction, the retinas were carefully dissected, cut into small pieces, and dissociated by enzymatic digestion. The single-cell suspensions, excluding dead cells (DAPI+), were analyzed by flow cytometry for MHC class II, CD80, CD86, and CD40 expression using fluorochrome-conjugated-specific antibodies. Data are representative of three independent animals for each disease model and timepoint, matched for disease grade. Only MHC class II^+^ cells are shown. A. Classical EAU, day 14. B. Classical EAU, day 21. C. Adoptive transfer EAU, day 14. (PPTX 2433 kb)

Additional file 6: **Figure S6.** Kinetics of MHC class II and hematopoietic cell marker expression on the three types of potential APCs during classical EAU and adoptive transfer EAU. Fourteen or 21 days after disease induction, retinas were carefully dissected, cut into small pieces, and dissociated by enzymatic digestion. The single-cell suspensions, excluding dead cells (DAPI+), were analyzed by flow cytometry for MHC class II, CD45, CD11b, and Ly6C expression using fluorochrome-conjugated specific antibodies. Data are representative of three independent animals for each disease model and timepoint, matched for disease grade. Data represented: Mean ± SEM. For each histogram, groups were compared using Kruskal-Wallis tests (all *p* values >0.05). A. Percentage of MHC class II^+^ cells in the retina during classical EAU or adoptive transfer (AT) EAU, at day 14 or day 21. B. Percentage of hematopoietic CD45^+^CD11b^+^ cells among MHC class II^+^ cells in the retina during classical EAU or AT EAU, at day 14 or day 21. C. MFI for MHC class II expression by hematopoietic or non-hematopoietic cells in the retina during classical EAU or AT EAU, at day 14 or day 21. D. Percentage of Ly6C^+^ cells among hematopoietic MHC class II^+^ cells in the retina during classical EAU or AT EAU, at day 14 or day 21. (PPTX 57 kb)

Additional file 7: **Figure S7.** Analysis of MHC class II expression in retinal wholemounts during adoptive transfer EAU. Three weeks after adoptive transfer, the eyes were collected and the whole retinas were dissected and stained for MHC class II (green) and endoglin (magenta) detection. Retinas from three independent animals were stained in one experiment. A. MHC class II and endoglin expression at the ora serrata. B. MHC class II and endoglin expression in the central retina. C. MHC class II and endoglin expression around the optic nerve. (PPTX 1345 kb)

## Abbreviations

Ag: Antigen
APC: Antigen-presenting cell
AT: Adoptive transfer
BRB: Blood-retinal barrier
CB: Ciliary body
CD: Cluster differentiation
CD11b: Itgam, integrin alpha M
CD31: PECAM-1, Platelet endothelial cell adhesion molecule 1
CD45: ptprc, protein tyrosine phosphatase, receptor type, C
CFA: Freund’s complete adjuvant
Cysltr1: Cysteinyl leukotriene receptor 1
DIC: Differential interference contrast
EAE: Experimental autoimmune encephalomyelitis
EAU: Experimental autoimmune uveitis
FACS: Fluorescence-activated cell sorting
FC: Flow cytometry
FDR: False discovery rate
FMO: Fluorescence minus one
FPKM: Fragments per kilobase of exon per million mapped reads
GFAP: Glial fibrillary acidic protein
IBA1: AIF1, allograft inflammatory factor 1
IF: Immunofluorescence
IRBP: Interphotoreceptor retinoid-binding protein
IRBP1-20: IRBP peptide 1–20
Lcn 2: Lipocalin 2
Log_2_FC: Log_2_ fold change
Ly6C: Lymphocyte antigen 6 complex
MHC: Major histocompatibility complex
NH: Non-hematopoietic
ONL: Outer nuclear layer
OS: Ora serrata
PFA: Paraformaldehyde
PTX: Pertussis toxin
RPE: Retinal pigment epithelium
SD: Standard deviation
SEM: Standard error of the mean
TCR: T cell receptor
T_H_: T helper lymphocyte
