# Supplementary figures and images for: MHC class II expression and potential antigen-presenting cells in the retina during experimental autoimmune uveitis
Source: J Neuroinflammation. 2017 Jul 18;14:136. doi: 10.1186/s12974-017-0915-5 (PMC5516361; doi:10.1186/s12974-017-0915-5)

## Slide 1
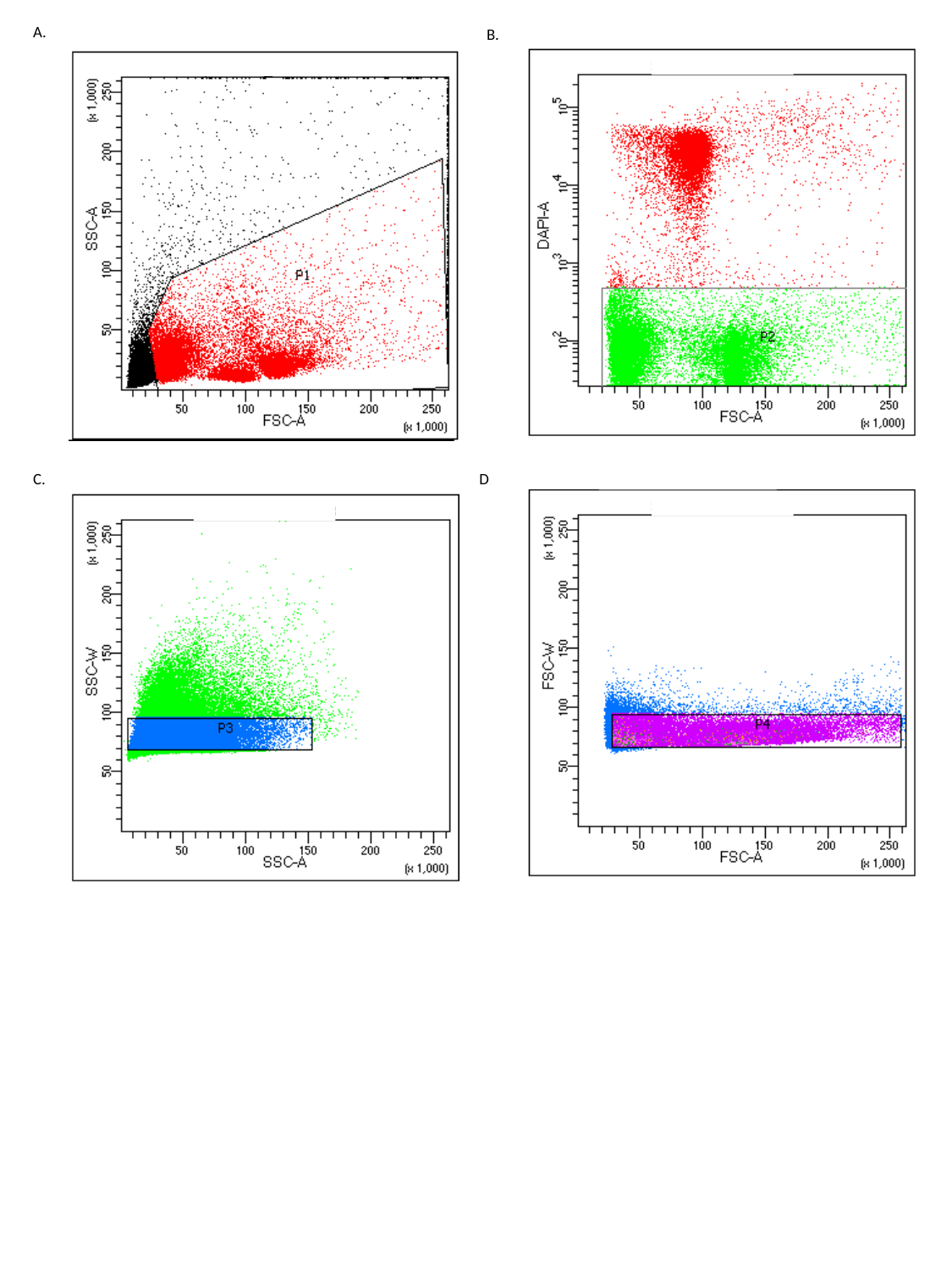

A.
B.
C.
D

Supplement: Supplementary file 1 — Figure S1. Complete gating strategy for flow cytometry experiments. Retinas were carefully dissected, cut into small pieces, and dissociated by incubation with Liberase DL and DNase I at 37 °C for 45 min. The single-cell suspensions were analyzed by flow cytometry. A. FSC versus SSC representation of the total cell population. The first gate was placed to exclude debris (P1). B. Within P1, Hoechst staining was used to exclude dead cells (P2). C. Within P2, doublets were excluded based on SSC (P3). D. Within P3, doublets were also excluded based on FSC (P4). All subsequent analyses were performed on cells gated in P4. (PPTX 106 kb) [file 12974_2017_915_MOESM1_ESM.pptx]

A.

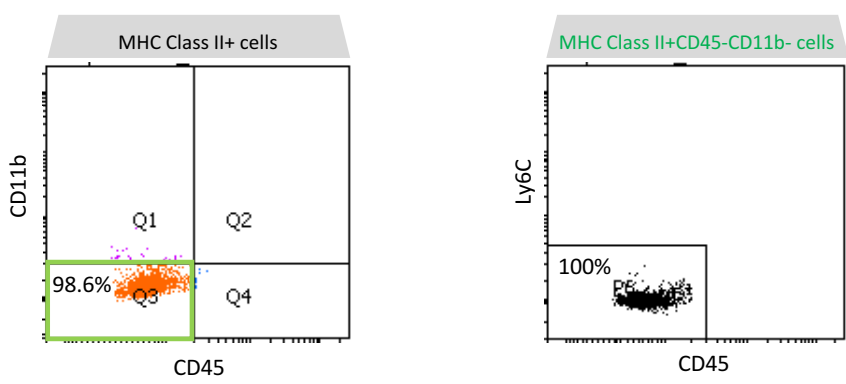

B.

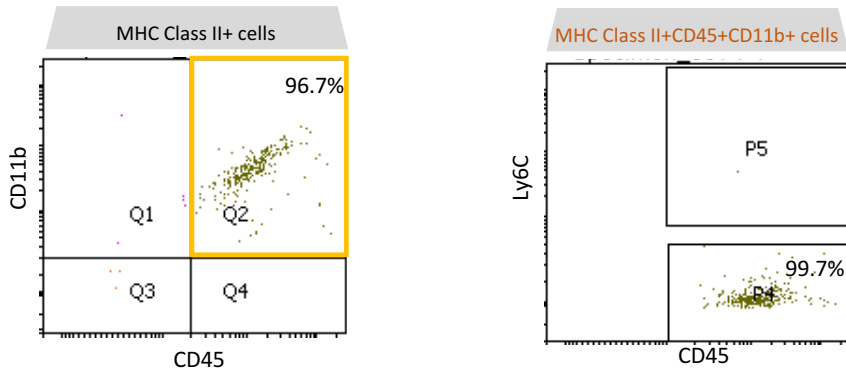

C.

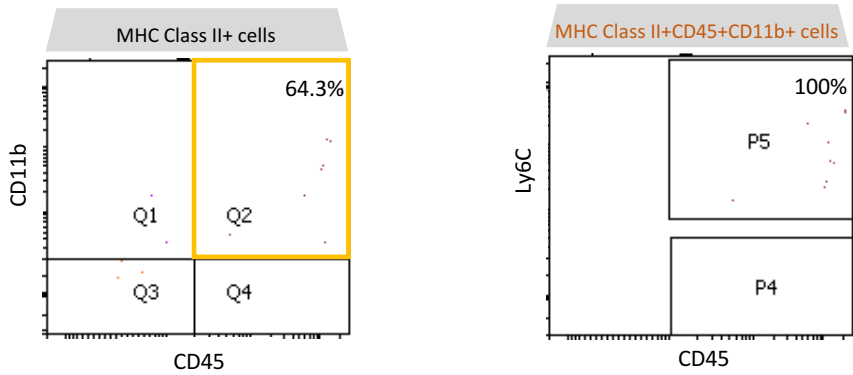

Supplement: Supplementary file 2 — Figure S2. Purity of the sorted cell populations. Three weeks after adoptive transfer, retinal single-cell suspensions were analyzed by flow cytometry and sorted into three different cell populations, MHC class II+CD45+CD11b+Ly6C+ (Plus), MHC class II+CD45+CD11b+Ly6C− (Minus), and MHC class II+CD45−CD11b−Ly6C− (NH) cells. Each sample was sorted from a pool of three mice. Sorted cells were then re-analyzed by flow cytometry to assess the purity of cell sorting. Although hampered by very low cell numbers due to death or adherence to tube of many cells between the two analyses, this figure illustrates the purity among live MHC class II+ cells. A. Sorted NH cells. B. Sorted Minus cells. C. Sorted Plus cells are too rare to allow reliable re-analysis (most re-analyzed Plus cells are found in the gate containing dead cells and debris). (PDF 354 kb) [file 12974_2017_915_MOESM2_ESM.pdf]
