# Supplementary material for: MHC class II expression and potential antigen-presenting cells in the retina during experimental autoimmune uveitis
Source: J Neuroinflammation. 2017 Jul 18;14:136. doi: 10.1186/s12974-017-0915-5 (PMC5516361; doi:10.1186/s12974-017-0915-5)
Supplement: Supplementary file 3 — Figure S3. MHC class II retinal expression is highly induced during classical EAU and adoptive transfer EAU, both during induction and at disease peak. Eye cryosections were stained for MHC class II (green) and IBA1 (red) detection 21 days after classical EAU induction (B), 14 days (C) or 21 days after adoptive transfer (AT) (D). Naive eyes were used as control (A). In each picture, quantification was made with the co-staining module of the Imaris 7.3 software. Each cell was counted individually. Results are expressed as the percentage of IBA1+ or MHCII+ single positive cells and IBA1+MHCII+ double-positive cells among the total of single and double-positive cells. The DIC image was added to better localize the RPE. A. MHC class II expression in naïve eyes. B. MHC class II expression during classical EAU at day 21. C. MHC class II expression during AT EAU at day 14. D. MHC class II expression during AT EAU at day 21. (PPTX 3600 kb) [file 12974_2017_915_MOESM3_ESM.pptx]

## Slide 1
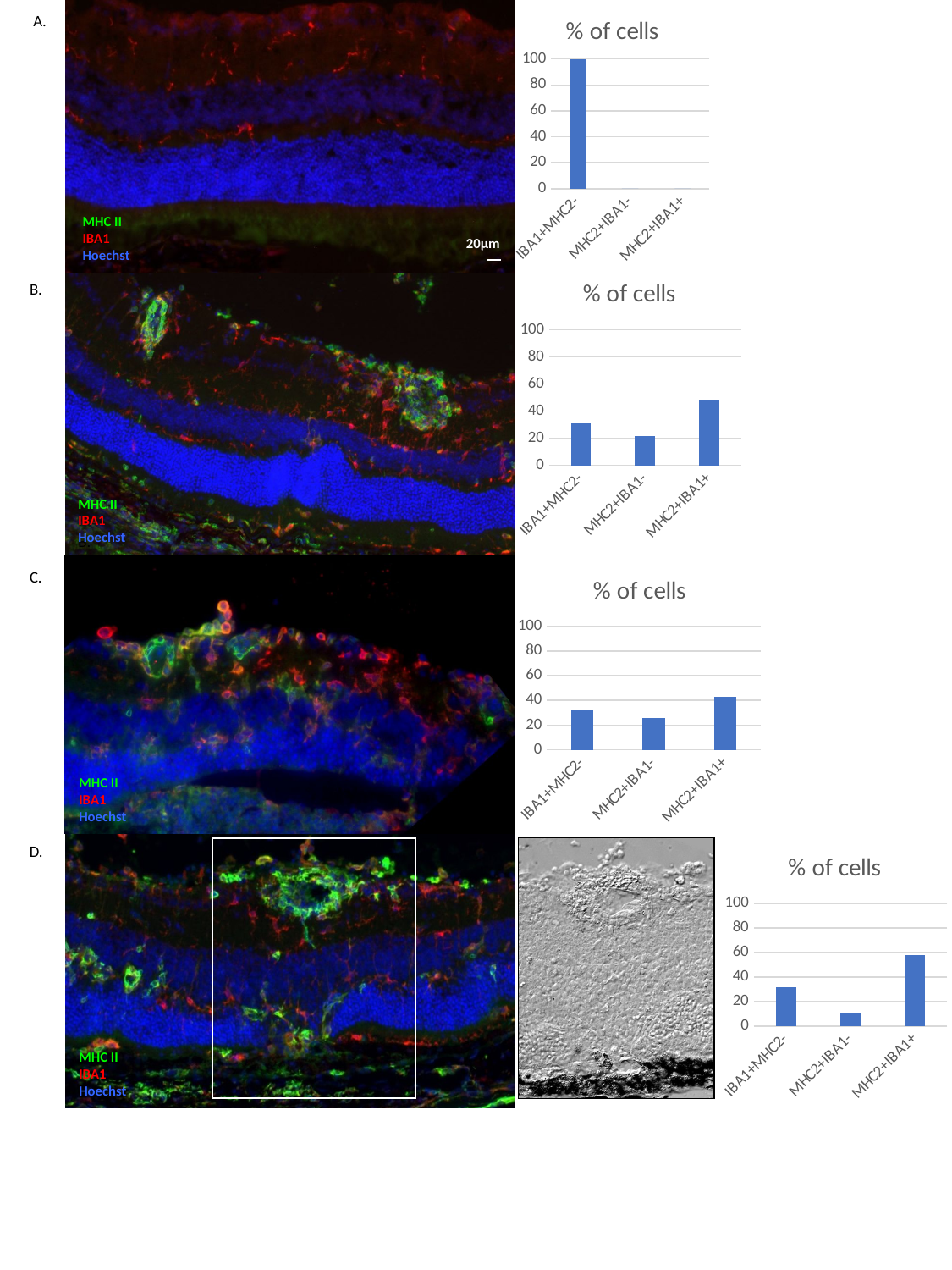

MHC II
IBA1
Hoechst
20µm
### Chart: % of cells
| Category | Fig1 |
|---|---|
| IBA1+MHC2- | 100.0 |
| MHC2+IBA1- | 0.0 |
| MHC2+IBA1+ | 0.0 |A.
### Chart: % of cells
| Category | Fig1 |
|---|---|
| IBA1+MHC2- | 30.95238095238095 |
| MHC2+IBA1- | 21.42857142857143 |
| MHC2+IBA1+ | 47.61904761904746 |B.
MHC II
IBA1
Hoechst
B.
C.
### Chart: % of cells
| Category | Fig1 |
|---|---|
| IBA1+MHC2- | 31.91489361702128 |
| MHC2+IBA1- | 25.53191489361702 |
| MHC2+IBA1+ | 42.55319148936159 |MHC II
IBA1
Hoechst
D.
### Chart: % of cells
| Category | |
|---|---|
| IBA1+MHC2- | 31.57894736842105 |
| MHC2+IBA1- | 10.52631578947368 |
| MHC2+IBA1+ | 57.89473684210526 |
MHC II
IBA1
Hoechst
