# Supplementary material for: MHC class II expression and potential antigen-presenting cells in the retina during experimental autoimmune uveitis
Source: J Neuroinflammation. 2017 Jul 18;14:136. doi: 10.1186/s12974-017-0915-5 (PMC5516361; doi:10.1186/s12974-017-0915-5)
Supplement: Supplementary file 4 — Figure S4. MHC class II expression in the retina during classical EAU. Three weeks after immunization, eye cryosections were prepared and stained for MHC class II (green) and IBA1 (red) or endoglin (magenta) detection. Cell nuclei were stained with Hoechst (blue). Each picture was chosen as representative of an experiment conducted on six or more animals. A. MHC class II and IBA1 expression. B. MHC class II and endoglin expression. (PPTX 7276 kb) [file 12974_2017_915_MOESM4_ESM.pptx]

## Slide 1
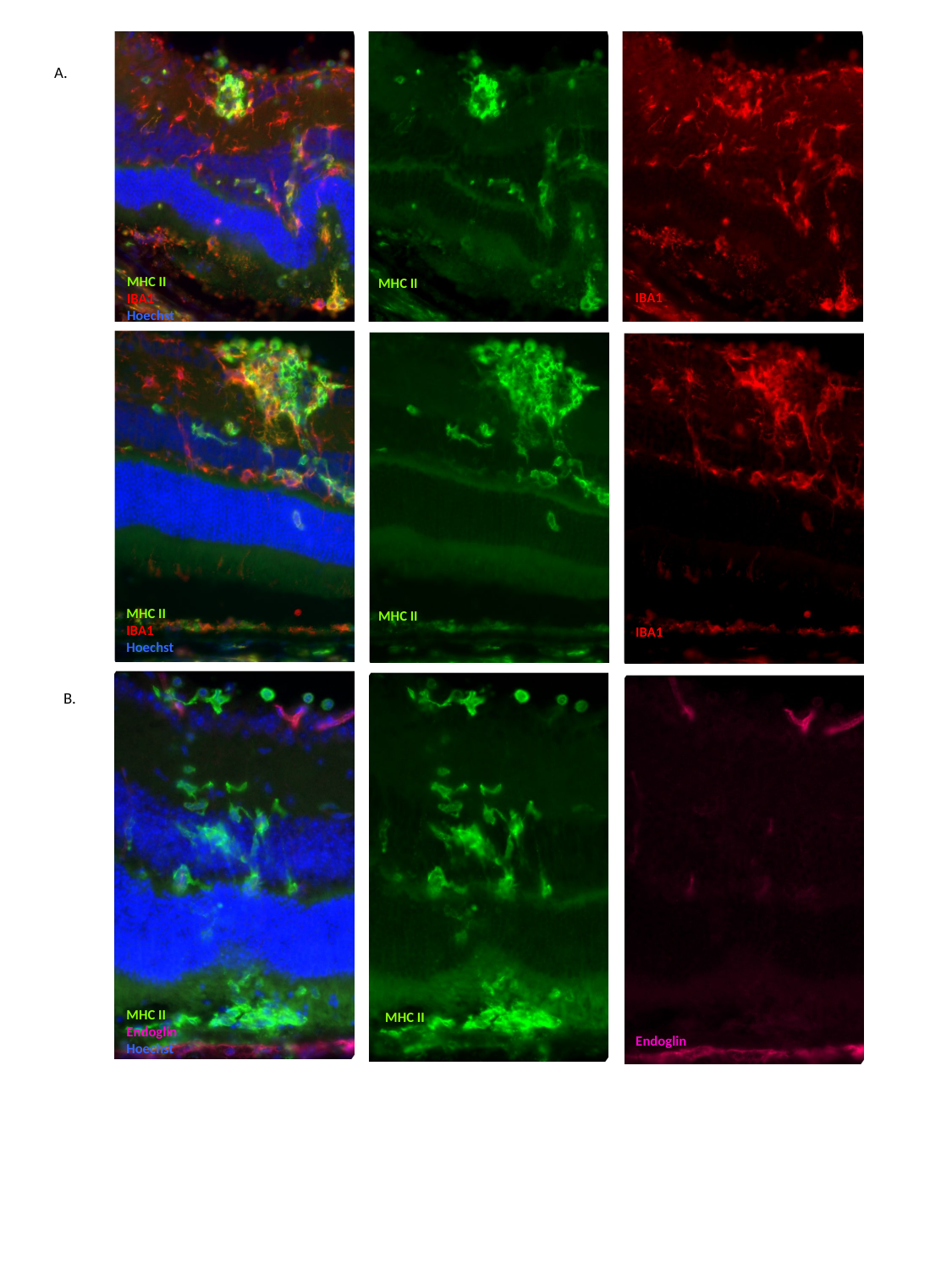

A.
IBA1
MHC II
IBA1
Hoechst
MHC II
MHC II
IBA1
Hoechst
IBA1
MHC II
B.
MHC II
Endoglin
Hoechst
MHC II
Endoglin
