# Supplementary material for: MHC class II expression and potential antigen-presenting cells in the retina during experimental autoimmune uveitis
Source: J Neuroinflammation. 2017 Jul 18;14:136. doi: 10.1186/s12974-017-0915-5 (PMC5516361; doi:10.1186/s12974-017-0915-5)
Supplement: Supplementary file 5 — Figure S5. Kinetics of co-stimulatory molecule expression by MHC class II± cells during classical EAU and adoptive transfer EAU. Fourteen or 21 days after disease induction, the retinas were carefully dissected, cut into small pieces, and dissociated by enzymatic digestion. The single-cell suspensions, excluding dead cells (DAPI+), were analyzed by flow cytometry for MHC class II, CD80, CD86, and CD40 expression using fluorochrome-conjugated-specific antibodies. Data are representative of three independent animals for each disease model and timepoint, matched for disease grade. Only MHC class II+ cells are shown. A. Classical EAU, day 14. B. Classical EAU, day 21. C. Adoptive transfer EAU, day 14. (PPTX 2433 kb) [file 12974_2017_915_MOESM5_ESM.pptx]

## Slide 1
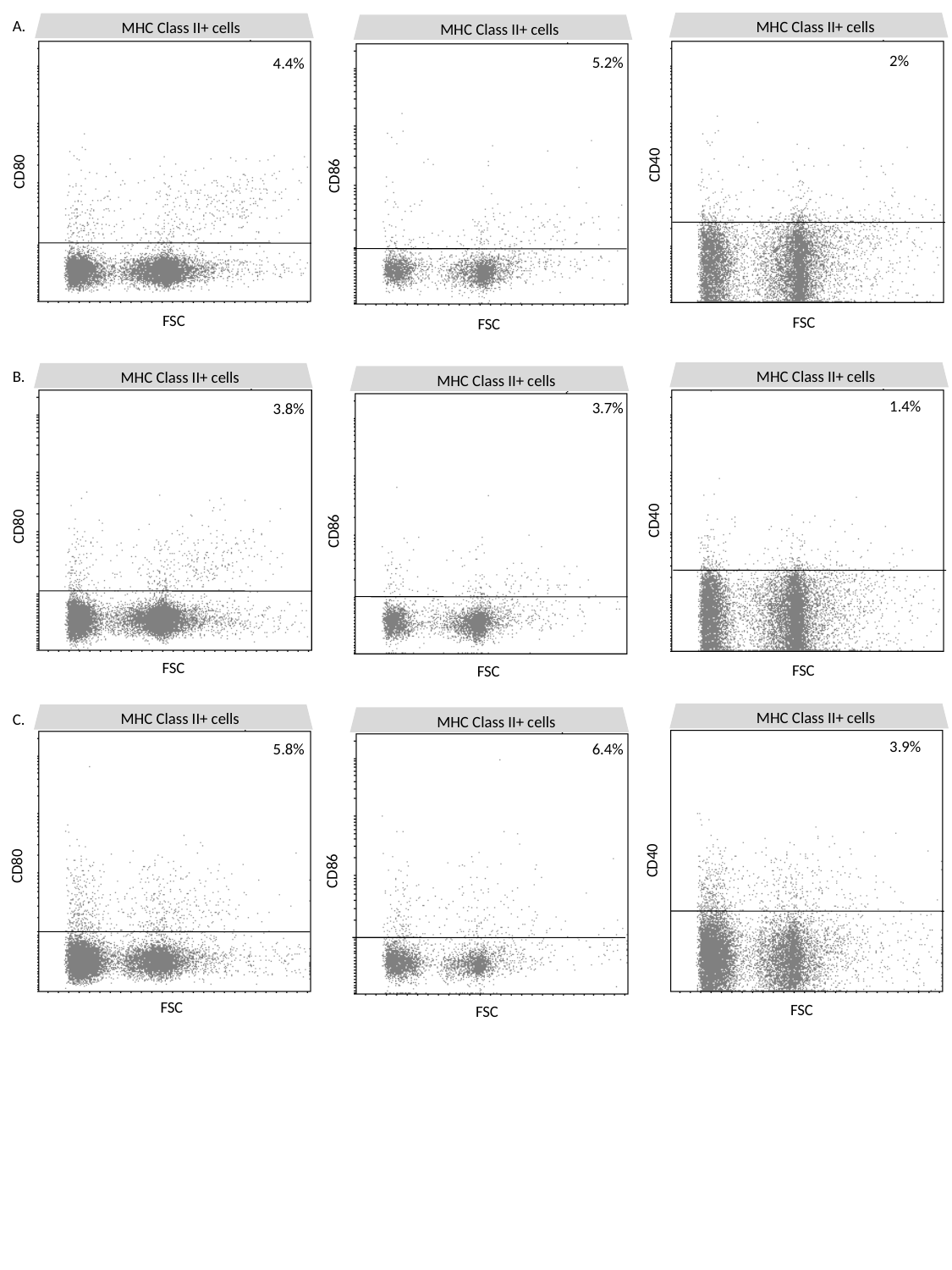

A.
MHC Class II+ cells
MHC Class II+ cells
MHC Class II+ cells
2%
5.2%
4.4%
CD40
CD80
CD86
FSC
FSC
FSC
MHC Class II+ cells
B.
MHC Class II+ cells
MHC Class II+ cells
1.4%
3.7%
3.8%
CD40
CD80
CD86
FSC
FSC
FSC
MHC Class II+ cells
MHC Class II+ cells
C.
MHC Class II+ cells
3.9%
6.4%
5.8%
CD40
CD80
CD86
FSC
FSC
FSC
