# Supplementary material for: MHC class II expression and potential antigen-presenting cells in the retina during experimental autoimmune uveitis
Source: J Neuroinflammation. 2017 Jul 18;14:136. doi: 10.1186/s12974-017-0915-5 (PMC5516361; doi:10.1186/s12974-017-0915-5)
Supplement: Supplementary file 6 — Figure S6. Kinetics of MHC class II and hematopoietic cell marker expression on the three types of potential APCs during classical EAU and adoptive transfer EAU. Fourteen or 21 days after disease induction, retinas were carefully dissected, cut into small pieces, and dissociated by enzymatic digestion. The single-cell suspensions, excluding dead cells (DAPI+), were analyzed by flow cytometry for MHC class II, CD45, CD11b, and Ly6C expression using fluorochrome-conjugated specific antibodies. Data are representative of three independent animals for each disease model and timepoint, matched for disease grade. Data represented: Mean ± SEM. For each histogram, groups were compared using Kruskal-Wallis tests (all p values >0.05). A. Percentage of MHC class II+ cells in the retina during classical EAU or adoptive transfer (AT) EAU, at day 14 or day 21. B. Percentage of hematopoietic CD45+CD11b+ cells among MHC class II+ cells in the retina during classical EAU or AT EAU, at day 14 or day 21. C. MFI for MHC class II expression by hematopoietic or non-hematopoietic cells in the retina during classical EAU or AT EAU, at day 14 or day 21. D. Percentage of Ly6C+ cells among hematopoietic MHC class II+ cells in the retina during classical EAU or AT EAU, at day 14 or day 21. (PPTX 57 kb) [file 12974_2017_915_MOESM6_ESM.pptx]

## Slide 1
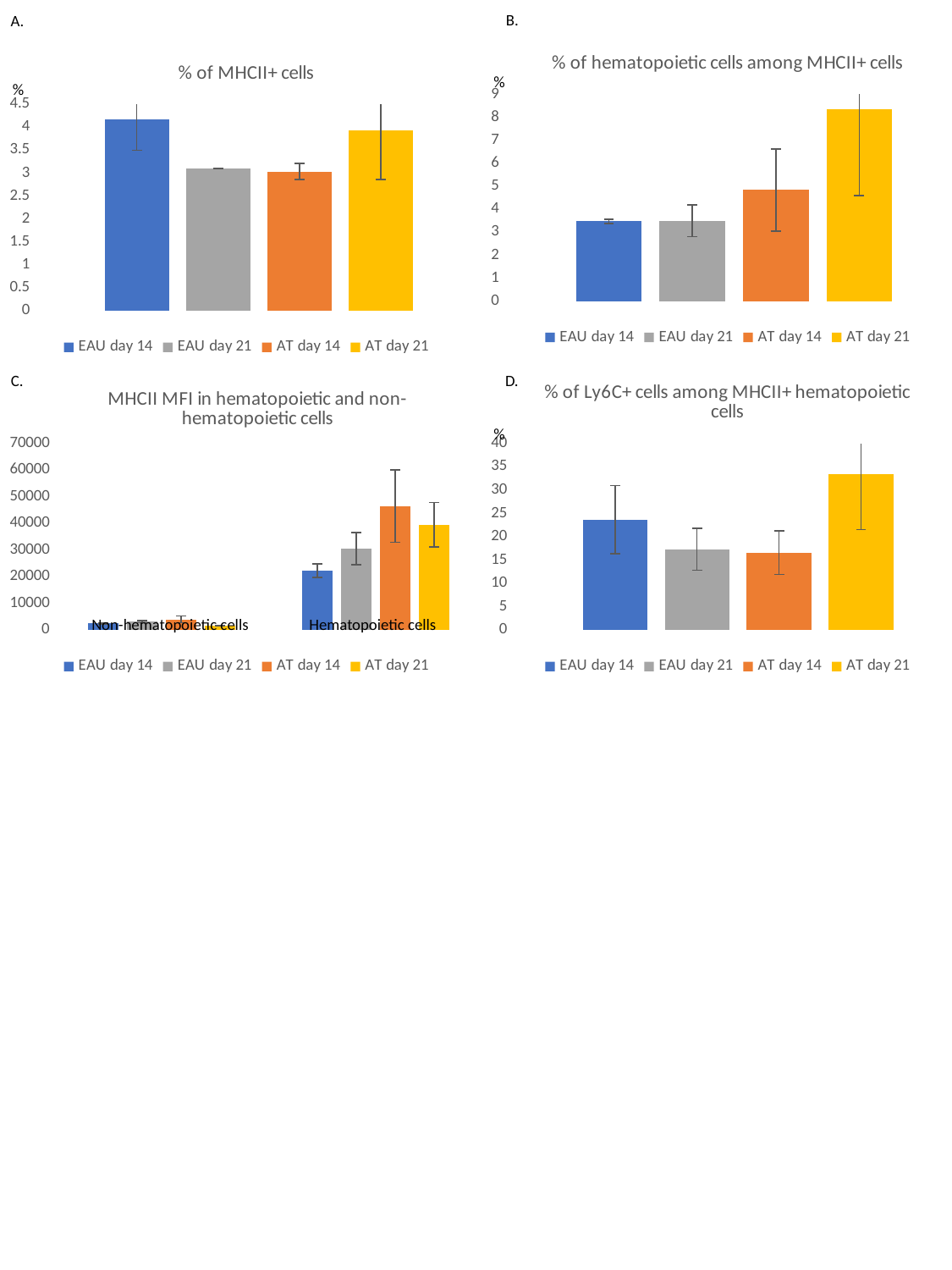

B.
A.
### Chart: % of hematopoietic cells among MHCII+ cells
| Category | EAU day 14 | EAU day 21 | AT day 14 | AT day 21 |
|---|---|---|---|---|
### Chart: % of MHCII+ cells
| Category | EAU day 14 | EAU day 21 | AT day 14 | AT day 21 |
|---|---|---|---|---|%
%
D.
C.
### Chart: MHCII MFI in hematopoietic and non-hematopoietic cells
| Category | EAU day 14 | EAU day 21 | AT day 14 | AT day 21 |
|---|---|---|---|---|
### Chart: % of Ly6C+ cells among MHCII+ hematopoietic cells
| Category | EAU day 14 | EAU day 21 | AT day 14 | AT day 21 |
|---|---|---|---|---|%
Non-hematopoietic cells
Hematopoietic cells
