# Supplementary material for: MHC class II expression and potential antigen-presenting cells in the retina during experimental autoimmune uveitis
Source: J Neuroinflammation. 2017 Jul 18;14:136. doi: 10.1186/s12974-017-0915-5 (PMC5516361; doi:10.1186/s12974-017-0915-5)
Supplement: Supplementary file 7 — Figure S7. Analysis of MHC class II expression in retinal wholemounts during adoptive transfer EAU. Three weeks after adoptive transfer, the eyes were collected and the whole retinas were dissected and stained for MHC class II (green) and endoglin (magenta) detection. Retinas from three independent animals were stained in one experiment. A. MHC class II and endoglin expression at the ora serrata. B. MHC class II and endoglin expression in the central retina. C. MHC class II and endoglin expression around the optic nerve. (PPTX 1345 kb) [file 12974_2017_915_MOESM7_ESM.pptx]

## Slide 1
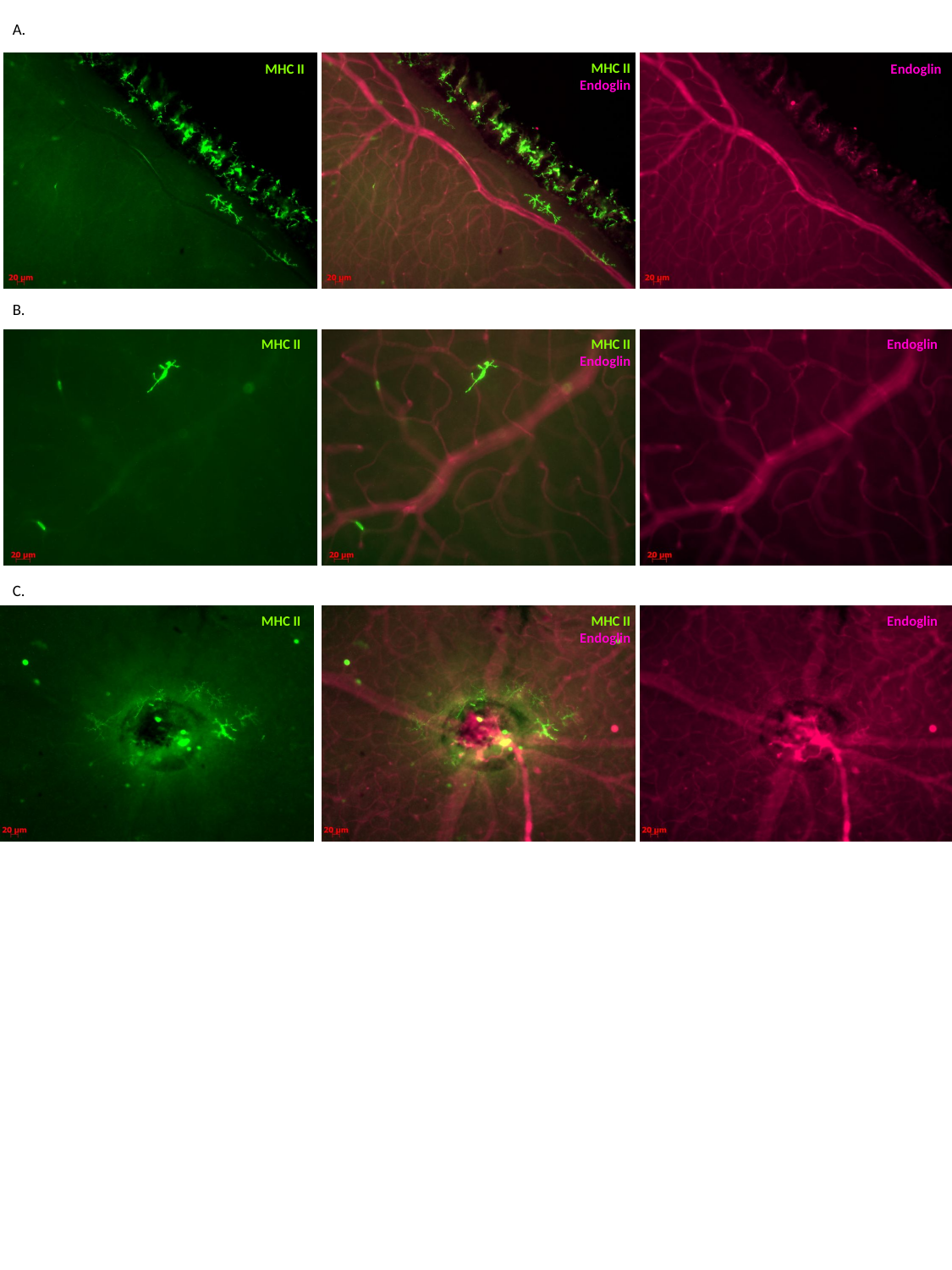

A.
MHC II
Endoglin
MHC II
Endoglin
B.
MHC II
Endoglin
MHC II
Endoglin
C.
MHC II
Endoglin
MHC II
Endoglin
